# Identification and structural validation of purine nucleoside phosphorylase from *Plasmodium falciparum* as a target of MMV000848

**DOI:** 10.1016/j.jbc.2023.105586

**Published:** 2023-12-21

**Authors:** Zara Chung, Jianqing Lin, Grennady Wirjanata, Jerzy M. Dziekan, Abbas El Sahili, Peter R. Preiser, Zbynek Bozdech, Julien Lescar

**Affiliations:** 1 School of Biological Sciences, Nanyang Technological University, Singapore, Singapore; 2 NTU Institute of Structural Biology, Experimental Medicine Building (EMB), Nanyang Technological University, Singapore, Singapore; 3 Antimicrobial Resistance Interdisciplinary Research Group, Singapore-MIT Alliance for Research and Technology Centre, Singapore, Singapore

**Keywords:** malaria, medicine for malaria ventures, drug resistance, target identification, cellular thermal shift assay, purine nucleoside phosphorylase, X-ray crystallography, enzyme inhibition

## Abstract

About 247 million cases of malaria occurred in 2021 with *Plasmodium falciparum* accounting for the majority of 619,000 deaths. In the absence of a widely available vaccine, chemotherapy remains crucial to prevent, treat, and contain the disease. The efficacy of several drugs currently used in the clinic is likely to suffer from the emergence of resistant parasites. A global effort to identify lead compounds led to several initiatives such as the Medicine for Malaria Ventures (MMV), a repository of compounds showing promising efficacy in killing the parasite in cell-based assays. Here, we used mass spectrometry coupled with cellular thermal shift assay to identify putative protein targets of MMV000848, a compound with an *in vitro* EC50 of 0.5 μM against the parasite. Thermal shift assays showed a strong increase of *P. falciparum* purine nucleoside phosphorylase (*Pf*PNP) melting temperature by up to 15 °C upon incubation with MMV000848. Binding and enzymatic assays returned a K_D_ of 1.52 ± 0.495 μM and an IC_50_ value of 21.5 ± 2.36 μM. The inhibition is competitive with respect to the substrate, as confirmed by a cocrystal structure of *Pf*PNP bound with MMV000848 at the active site, determined at 1.85 Å resolution. In contrast to transition states inhibitors, MMV000848 specifically inhibits the parasite enzyme but not the human ortholog. An isobologram analysis shows subadditivity with immucillin H and with quinine respectively, suggesting overlapping modes of action between these compounds. These results point to *Pf*PNP as a promising antimalarial target and suggest avenues to improve inhibitor potency.

The majority of over 619,000 deaths due to malaria occurs in sub-Saharan Africa with infants under 5 years paying the highest toll to the disease (https://www.who.int/publications/i/item/9789240064898). While malaria can be caused in humans by five species of *Plasmodium* merozoites with tropism for human erythrocytes: *Plasmodium falciparum, Plasmodium vivax, Plasmodium ovale, Plasmodium malariae*, and *Plasmodium knowlesi*, *P. falciparum* accounts for more than 98% of the observed mortality (1). Current measures to combat malaria include mosquito vector-control strategies, use of mosquito nets, and implementation of artemisinin combination therapies (2). Genetic resistance is gradually increasing against artemisinin (3, 4), with the first incidence reported in Cambodia over 20 years ago (5). Between 2014 and 2016, partial resistance to artemisinin was also noted in several African countries, raising serious public health concerns, because of the massive disease burden already suffered by this continent (6, 7). With a view to expand the drug armamentarium against malaria, several global initiatives were started to assemble lead compound libraries that showed promising antiparasite activities in cell-based phenotypic assays (8). These chemical repositories include the GlaxoSmithKline Tres Cantos antimalaria set (9) and the Medicines for Malaria Venture (MMV) (10).

The MMV “Malaria Box” contains about four hundred diverse drug-like compounds identified in phenotypic screens against *P. falciparum*, which were distributed to research laboratories for a concerted open-access effort for drug discovery against malaria (10). While phenotypic screens provide indispensable libraries of leads to combat malaria, in many cases little is known about the precise mechanism of action employed by these compounds to kill the parasite. A better knowledge of their targets is required to improve the selectivity and potency of promising hits and several methods were devised for target deconvolution. These comprise *in vitro* selection of resistant parasites grown in the presence of inhibitory compounds, associated with genome sequencing (11) and the use of clickable probes (12). Recently, a powerful label-free tool for identifying malaria drug targets was designed, by repurposing the mass spectrometry-cellular thermal shift assay (MS-CETSA) (13), a proteomics method initially designed for anticancer drug targets identification (14).

Several molecular drug targets from the *Plasmodium* proteome have been proposed and prioritized based on a number of criteria including genetic validation, resistance potential, druggability, availability of an activity assay, and structural information (15). This constitutes a paradigm shift from the conventional phenotypic approach that has been used so far, towards a more target-based approach. Along this line, that the malaria parasites lacks *de novo* purine biosynthesis indicates potential for targeting this pathway (16, 17) that comprises (i) purine nucleoside phosphorylase, (ii) hypoxanthine-guanine-xanthine-phosphoribosyl transferase, and (iii) adenosine deaminase (17, 18). Purines are obtained by the parasite from the host erythrocyte mostly *via* hypoxanthine, the major purine base salvaged by *Plasmodium*, through metabolizing adenosine *via* the sequential action of adenosine deaminase and purine nucleoside phosphorylase (*Pf*PNP) (18, 19). *Pf*PNP catalyzes the phosphorylation of inosine and guanosine to ribose-1 phosphate and hypoxanthine or guanine, respectively (Fig. 1*A*). Since hypoxanthine synthesized through this purine salvage pathway constitutes a major source of purines for the parasite, *Pf*PNP is essential for parasite survival (19, 20, 21, 22, 23, 24, 25).

**Figure 1:**
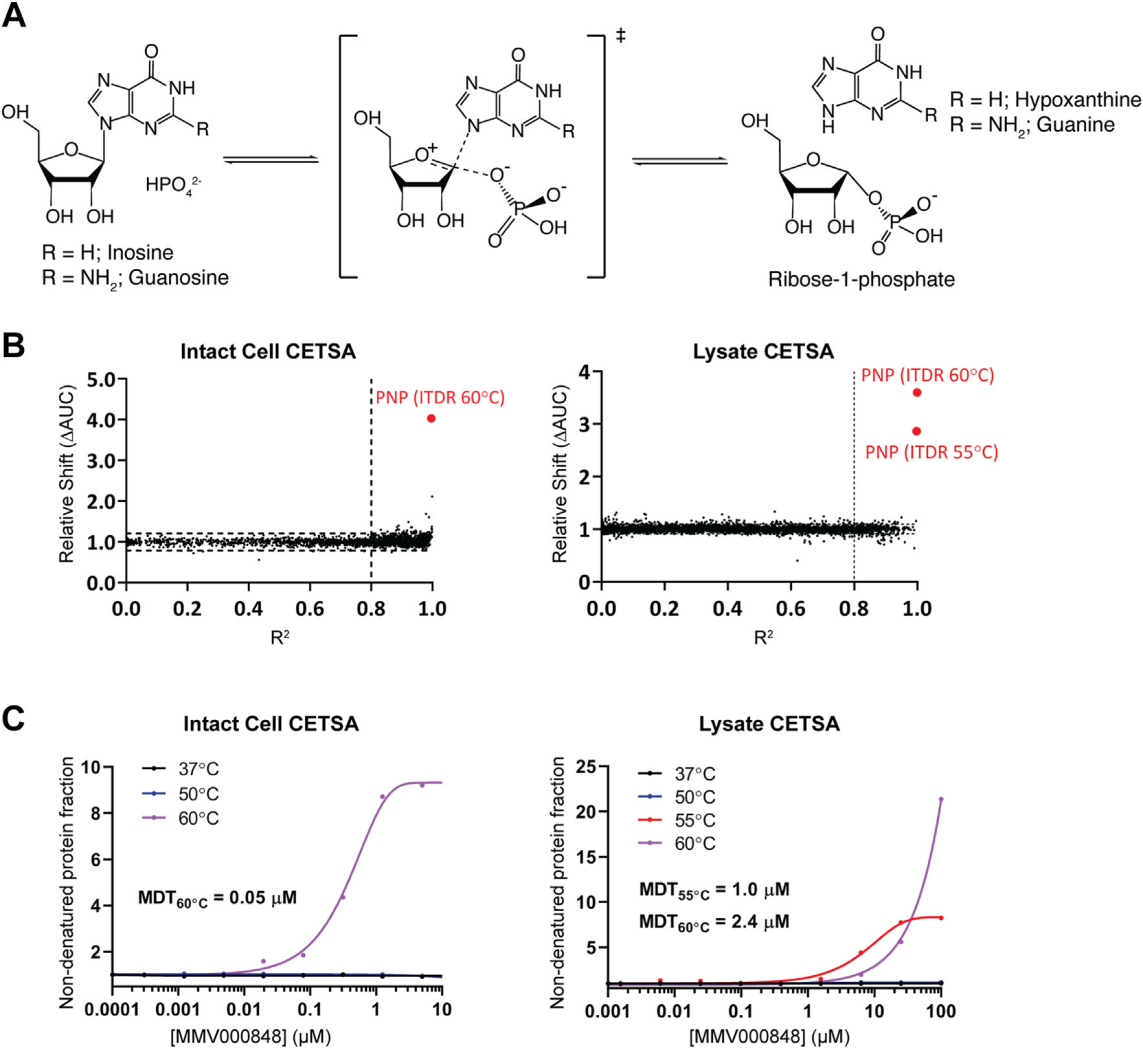
**Reaction catalyzed by PfPNP and identification of PfPNP as a target of MMV000848.***A*, *Pf*PNP catalyzes the conversion of inosine into ribose-1-phosphate and hypoxanthine, the major precursor for the purine salvage pathway. Guanosine is a less favored substrate that leads to guanine formation by *Pf*PNP. The middle panel depicts the ribo-cationic transition state (shown in *brackets*) (25). *B*, scatter plots representing the distribution of protein stabilization obtained from intact cell MS-CETSA, plotted as a function of R2 value (goodness of curve fit) against ΔAUC (area under the curve of heat-challenged sample normalized against non-denaturing 37 °C control) for all proteins detected in the assay. The cut-offs used: three times of median absolute deviation (MAD) of ΔAUC in each dataset (MAD × 3) and R2 = 0.8 are indicated on the graph. The parasite PNP (*red dots*) is the most stabilized protein of the proteome. The complete list of *Plasmodium falciparum* proteins stabilized by MMV000848 can be found in Tables 1 and 2. *C*, thermal stabilization profiles of *Pf*PNP obtained by CETSA from intact cells (*left*) and cell lysate (*right*). Stabilization under thermal challenges (colored) is plotted relative to no-drug control with non-denaturing control (37 °C) plotted in *black*. Minimal dose threshold (MDT) values stated are from heat treatment temperature, where thermal stabilization was observed. MMV, Medicine for Malaria Ventures; MS-CETSA, mass spectrometry coupled with cellular thermal shift assay; *Pf*PNP, *Plasmodium falciparum* purine nucleoside phosphorylase.

The *in vivo* relevance of *Pf*PNP as target was shown by Cassera *et al.* (2011) (26) who tested the efficacy of DADMe-immucillin G (or “BCX-4945”) in *Aotus* primates, an animal model for *P. falciparum* infections. Oral administration of BCX-4945 over a week resulted in parasite clearance in the animals (26). Pioneering studies by the Schramm group on *Homo sapiens* PNP (*Hs*PNP) and *Pf*PNP led to the identification of a transition-state analog immucillin H (ImmH) that showed 56 pM dissociation constant to *Hs*PNP. This was followed by the development of several analogs of immucillin (18). Remarkably, ImmH also binds *Pf*PNP with a K_D_ of 860 pM (27, 28) and kills *P. falciparum* cultured in human erythrocytes with an IC_50_ of 35 nM (22, 29). Addition of a thio-methyl group at the 5′ carbon of ImmH to produce 5′ methylthio-Immucillin H (MT-ImmH) shifted the specificity towards the parasite enzyme over the human analog by a factor of 112, although this inhibitor still inhibits both the human and parasite enzymes (28, 30). This demonstrated that parasite-specific active site inhibitors could in principle be designed, despite similarity with the human enzyme. Using the MS-CETSA approach, *Pf*PNP was shown to be targeted by quinine with a reported K_i_ of 138 nM while mefloquine was less potent (31). Both quinoline drugs bind to the enzyme active site and are competitive inhibitors with respect to the substrate, lending support to the hypothesis that *Pf*PNP plays a role in their mechanism of action (31).

Here, over the course of a systematic malaria drug targets deconvolution campaign using MS-CETSA, we found that *Pf*PNP is thermostabilized by compound MMV000848 from the malaria box (10). We show that MMV000848 strongly engages *Pf*PNP both in cell lysate and in intact cell-based CETSA assays. We report biophysical, enzymatic, and structural data including a cocrystal structure of MMV000848 bound at the active site of *Pf*PNP at a 1.85 Å resolution. An isobologram analysis demonstrates subadditivity between MMV000848 and other *Pf*PNP inhibitors, including immucillin H and quinine, consistent with a degree of overlap in the mechanism of action used by these drugs. Taken together, these data show a competitive mode of inhibition of *Pf*PNP by MMV000848 which is not observed for *Hs*PNP and should help develop novel antimalarial compounds targeting *Pf*PNP with improved specificity and potency.

## Results

### MS-CETSA identifies PfPNP as a target of MMV000848

Here we employed MS-CETSA to interrogate the mechanism of action of MMV000848. We used cellular lysates from *in vitro* blood-stage *P. falciparum* parasites to study direct drug–target interactions (“lysate”). In order to study drug-target engagements in an intact cell context, we also used live erythrocytes infected by *P. falciparum* (“intact cell”). We carried out an Iso-Thermal Dose Response (ITDR) assay. In this implementation of MS-CETSA, protein thermal stabilization is monitored at a single temperature in the presence of a range of drug concentrations (∼1 nM–10 μM) covering the *in vitro* IC50 value of MMV000848 of 0.5 μM. The complete list of *P. falciparum* proteins stabilized by MMV000848 can be found in Tables 1 and 2.

**Table 1:** Protein hits identified from Lysate CETSA

PlasmoDB ID	Target name	Heat challenge (°C)	MDT (nM)
PF3D7_0513300	Purine nucleoside phosphorylase	55	1047.2
		60	2425.8
PF3D7_1358800	40S ribosomal protein S15	60	48,373.9
PF3D7_0210100	60S ribosomal protein L37ae (putative)	55	25,032.5
PF3D7_0531100	Conserved *Plasmodium* protein, unknown function	55	4798.9
PF3D7_1360800	Falcilysin	60	11,089.5
PF3D7_1416500	NADP-specific glutamate dehydrogenase	60	29.8

Analysis was conducted at 50 °C, 55 °C, and 60 °C across 1906, 1787, and 1676 proteins, respectively.

**Table 2:** Protein hits identified from Intact Cell CETSA

PlasmoDB ID	Target name	Heat challenge (°C)	MDT (nM)
PF3D7_0513300	Purine nucleoside phosphorylase	60	22.23
PF3D7_0110800	Transcription initiation factor TFIIB putative	60	196.53
PF3D7_0204700	Hexose transporter	50	316.49
PF3D7_0311300	Phosphatidylinositol 3- and 4-kinase putative	60	985.85
PF3D7_0312800	60S ribosomal protein L26 putative	50	189.79
PF3D7_0407800	Conserved Plasmodium protein unknown function	60	609.72
PF3D7_0409400	Chaperone protein DnaJ	60	1605.60
PF3D7_0505500	DNA mismatch repair protein MSH6 putative	60	383.36
PF3D7_0505700	Conserved Plasmodium membrane protein unknown function	60	3846.84
PF3D7_0526200	ADP-ribosylation factor GTPase-activating protein putative	50	1290.89
PF3D7_0611600	Basal complex transmembrane protein 1	60	351.65
PF3D7_0710600	60S ribosomal protein L34	50	339.18
PF3D7_0819600	Conserved Plasmodium protein unknown function	50	1440.99
PF3D7_0822900	Conserved Plasmodium protein unknown function	60	1137.80
PF3D7_0914700	Major facilitator superfamily-related transporter putative	50	479.60
PF3D7_0919900	Regulator of chromosome condensation-PP1-interacting protein	60	620.45
PF3D7_1014900	Conserved Plasmodium protein unknown function	60	1221.07
PF3D7_1031600	Conserved Plasmodium protein unknown function	60	19,500.89
PF3D7_1037300	ADP/ATP transporter on adenylate translocase	50	240.00
PF3D7_1109900	60S ribosomal protein L36	50	150.27
PF3D7_1116800	Heat shock protein 101	50	817.34
PF3D7_1121600	Exported protein 1	50	982.29
PF3D7_1124700	GrpE protein homolog mitochondrial putative	60	735.92
PF3D7_1129000	Spermidine synthase	50	265.77
PF3D7_1134000	Heat shock protein 70	60	994.72
PF3D7_1211900	Non-SERCA-type Ca2+ -transporting P-ATPase	50	430.33
PF3D7_1341300	60S ribosomal protein L18-2 putative	50	534.94
PF3D7_1352800	Vacuolar fusion protein MON1 putative	60	384.28
PF3D7_1360800	Falcilysin	60	749.91
PF3D7_1417500	H/ACA ribonucleoprotein complex subunit 4 putative	60	784.24
PF3D7_1431700	60S ribosomal protein L14 putative	50	665.11
PF3D7_1434800	Mitochondrial acidic protein MAM33 putative	60	267.45
PF3D7_1471100	Exported protein 2	50	1188.97

Analysis was conducted at 50 °C and 60 °C across 1166 and 825 proteins, respectively.

Falcilysin (PF3D7_1360800) and PNP (PF3D7_0513300) appeared as two proteins exhibiting high-confidence stabilization levels among a total of 2157 and 2032 *P. falciparum* proteins detected simultaneously in both datasets (Tables 1 and 2). Having a large hydrophobic pocket, falcilysin is a promiscuous target from the hemoglobin digestion pathway that is able to engage many drugs including several quinolines but also investigational drugs such as MK-4815, a compound developed by Merck. A detailed analysis of the interactions formed between the active site chamber of falcilysin and these various drugs and drug candidates is given in a separate manuscript (32). Scatter plots representing the combined target selection matrix from different heat challenge temperatures are displayed in Figure 1*B*. A clear dose-dependent stabilization of *Pf*PNP was observed upon MMV000848 treatment (Fig. 1*C*), with minimum dose threshold (minimum concentration needed to achieve thermal stabilization) ranging from ∼0.05 μM in intact cells and ∼1.0 and 2.4 μM for cell lysate at 55 °C and 60 °C heat challenge, respectively. (Fig. 1*C*). Although the precise origin of the ∼50 fold lower value of the minimum dose threshold (60 °C) observed in the intact-cell CETSA compared to the lysate CETSA is unknown, the difference might be due to a MMV000848 accumulation or concentration effect in the parasite. Next, we decided to explore the interactions between MMV000848 and *Pf*PNP in more detail.

### MMV000848 binds to PfPNP and inhibits its enzyme activity *in vitro* but does not bind the human ortholog

**Figure 2:**
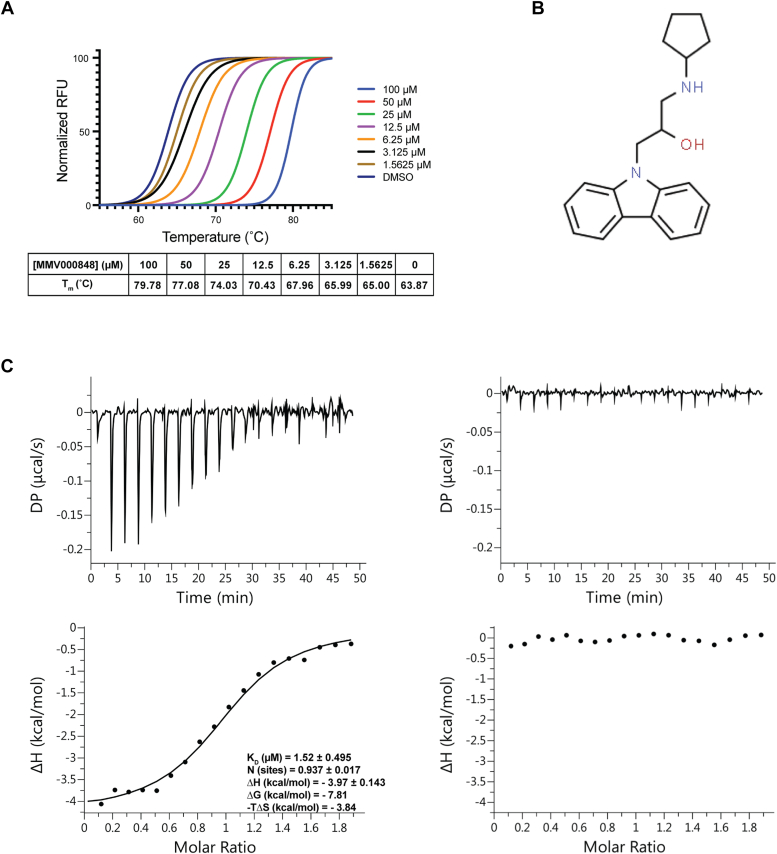
**MMV000848 shows direct binding to *Pf*PNP but not to the human ortholog.***A*, normalized melting curve from DSF analysis of *Pf*PNP stabilization by MMV000848 carried out with a concentration of 15 μM of *Pf*PNP and various concentrations of MMV000848 (100 μM to 1.25625 μM). Each data point was measured in triplicates. The melting temperature Tm measured from Boltzmann sigmoidal analysis indicated dose-dependent stabilization of *Pf*PNP. *B*, schematic structure of MMV000848. This compound from the MMV “malaria box” has an EC50 of 0.5 μM against the parasite. It is composed of a 5-carbon ring linked to a carbazole aromatic group composed of three fused rings. *C*, isothermal titration calorimetry measurement of the interaction between MMV000848 and *Pf*PNP (*left*) or *Hs*PNP (*right*). Top graph shows injection peaks with the area of each peak proportional to the heat released from each injection. Bottom profile shows the binding isotherms. The binding affinity, stoichiometry, and thermodynamic parameters for the interaction between MMV000848 and PfPNP are indicated. No interaction was observed between MMV000848 and *Hs*PNP, indicating specificity of the inhibitor for the *Plasmodium* enzyme. DSF, differential scanning fluorimetry; MMV, Medicine for Malaria Ventures; *Pf*PNP, *Plasmodium falciparum* purine nucleoside phosphorylase.

To validate the interaction between *Pf*PNP and MMV000848 identified *via* MS-CETSA, we first determined the stabilizing effect against thermal denaturation imparted to the recombinant protein by this compound using differential scanning fluorimetry. We observed concentration-dependent stabilization of *Pf*PNP similar to that of the positive control ImmH (Fig. S1) with a significant increase in the melting temperature T_m_ of 15.9 °C when the enzyme was incubated with a concentration of 100 μM of MMV000848 (Fig. 2*A*). Next, determination of the dissociation constant was done using isothermal titration calorimetry (ITC) returning a K_D_ of 1.52 ± 0.49 μM for a 1:1 interaction between MMV000848 and each *Pf*PNP monomer (Fig. 2*C*). This value corresponds to a 7-fold higher affinity than the previously identified *Pf*PNP active site inhibitor mefloquine (K_D_ = 11 μM) (31) while quinine, which also binds to the enzyme active site, has a reported K_D_ of 65 nM (although a biphasic fitting was performed to obtain the latter value, see ref. 32 and discussion). The thermodynamic parameters with ΔG of −7.91 kcal/mol reveal both significant enthalpic (ΔH = −3.97 kcal/mol) and entropic contributions (−TΔs = −3.84 kcal/mol), which are in line with the observation of several hydrogen bonds and water-mediated interactions, as discussed in the structural section. In contrast, no interaction could be detected between MMV000848 and the human enzyme indicating strict specificity of the inhibitor for the parasite enzyme (Fig. 2*C* right panel).

**Figure 3:**
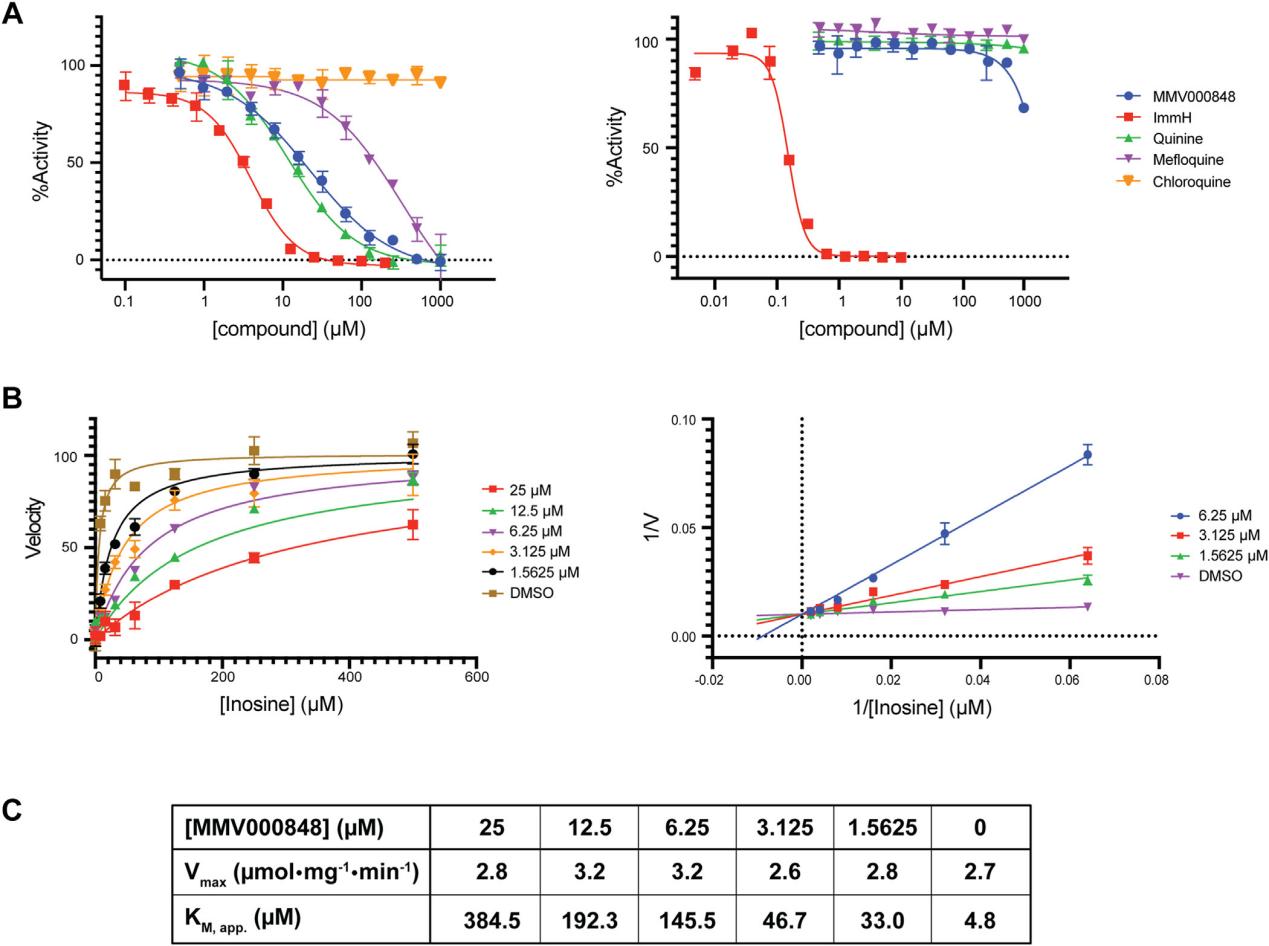
***In vitro* enzymatic assay and mode of inhibition.***A*, a concentration of 100 nM of either *Pf*PNP (*left panel*) or *Hs*PNP (*right panel*) was incubated with 60 mU/ml xanthine oxidase and 200 μM inosine in the presence of various concentrations of the following inhibitors: MMV000848, quinine, mefloquine, and chloroquine. For *Hs*PNP, the range of inhibitor concentrations used were as follows: 1 mM to 488.28 nM for MMV000848; 10 μM to 4.88 nM for immucillin H (ImmH). The IC_50_ values were determined from sigmoidal, four-parameter logistic analysis. For *Pf*PNP, IC_50_ values are as follows: MMV000848: 21.49 μM, ImmH: 3.824 μM, quinine: 11.33 μM, and mefloquine: 327.3 μM. For *Hs*PNP, the IC_50_ value of ImmH is 157.1 nM. *B*, *Pf*PNP enzyme kinetics parameters were measured to determine Ki (using competitive inhibition model) as well as values of V_max_ and apparent K_M,app_ (using Michaelis Menten model). Fixed concentrations of 50 nM of *Pf*PNP, 60 mU/ml xanthine oxidase, and a range of concentrations from 500 μM to 7.8125 μM of inosine were used, in the presence of 50 μM to 1.5626 μM of MMV000848. The double-reciprocal Lineweaver-Burk plot (*right panel*) indicates competitive inhibition (see text for details). *C*, *Pf*PNP kinetic parameters were determined from Michaelis Menten analysis. Measurements of K_M,app_ (apparent K_M_) of inosine was made in the presence of various concentrations of MMV000848. The experiment was carried out in triplicates. MMV, Medicine for Malaria Ventures; *Pf*PNP, *Plasmodium falciparum* purine nucleoside phosphorylase.

Enzyme activity was measured by following the conversion of inosine to hypoxanthine (29, 33). A fixed concentration of 100 nM of *Pf*PNP was incubated with various concentrations of MMV000848. Previously identified inhibitors of *Pf*PNP: mefloquine, quinine, and ImmH (also named “BCX-1777”) (22, 28, 29, 30, 34) were used for comparison, and chloroquine as a negative control (Fig. 3). In this assay, MMV000848 inhibited *Pf*PNP activity with a 50% inhibitory concentration, IC_50_ of 21.49 μM, which is intermediate between the IC_50_ values of mefloquine (IC_50_ = 327.3 μM) and quinine (K_i_ =11.33 μM) (Fig. 3*A*, left panel and Table S1). Accordingly, the K_i_ of MMV000848 was 454.90 nM (Fig. 3*B*, left panel and Table S1), which is also comprised between previously reported values for quinine (K_i_ = 138 nM) and mefloquine (5.9 μM). This ranking in inhibition potency agrees with ITC results where MMV000848 has a binding affinity intermediate between mefloquine and quinine (31). In order to examine the mode of inhibition employed by MMV000848 with respect to the inosine substrate, a Lineweaver-Burk double reciprocal plot was computed (Fig. 3*B*, right panel). Increasing concentrations of compound MMV000848 only resulted in minimal changes of V_max_ values of *Pf*PNP, while an increase of apparent K_M,app_ values for inosine was observed (Fig. 3*C*). This assay indicates that MMV000848 behaves as a competitive inhibitor of the interaction between *Pf*PNP and inosine and is therefore likely to bind to the enzyme active site (Fig. 3*B*). To investigate potential toxicity due to off-target inhibition, we also carried out enzymatic inhibition assay with purified recombinant *Hs*PNP in the presence of MMV000848, mefloquine, quinine, and ImmH. The transition state analog ImmH showed an IC_50_ of 157.1 nM, while MMV000848, mefloquine, and quinine did not inhibit the human ortholog (Fig. 3*A*, right panel).

### Crystal structure of the *Pf*PNP–MMV000848 complex

**Figure 4:**
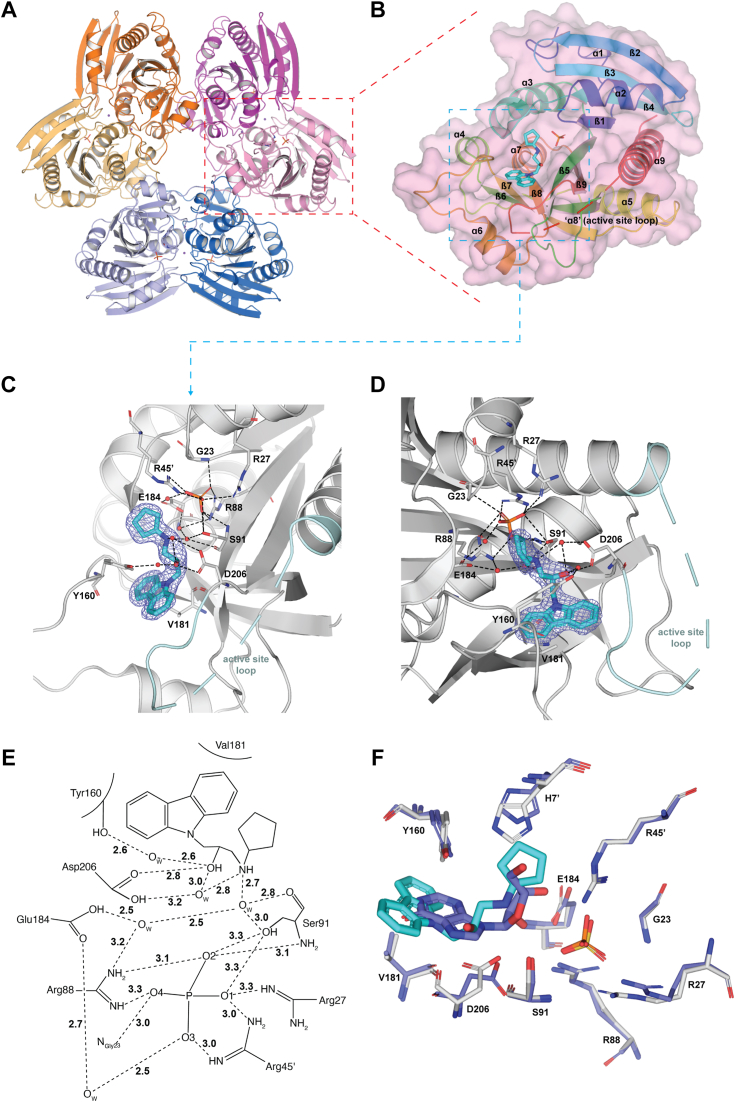
**Structure of *Pf*PNP-MMV000848 and comparison with the native enzyme-substrate complex.***A*, *Pf*PNP hexameric structure with each dimer colored differently (as *dark* and *lighter tone*). The bound MMV000848 inhibitor and phosphate ions are shown as sticks. *B*, transparent surface representation of the *Pf*PNP monomer with MMV000848 (*cyan sticks*) and phosphate (*orange sticks*) molecules bound at the active site. The *Pf*PNP main chain is shown as ribbons colored from *blue* (N-terminus) to *red* (C-terminus). Several elements of secondary structures discussed in the text are indicated. *C* and *D*, magnified views (separated by about 90° rotation) of the active site highlighting residues interacting with MMV000848. A F_0_–F_c_ difference Fourier electron density residual map with MMV000848 omitted from the phase calculation is contoured at a level of 3.0 σ and displayed as a slate mesh. The active site loop is colored in *cyan* and missing atoms are represented by *dashed lines*. Hydrogen bonds are depicted as *dashes* and water molecules mediating inhibitor–protein interactions as *red spheres*. *E*, 2D diagrammatic representation of interactions between *Pf*PNP and MMV000848 and the phosphate. Dashes represent hydrogen bonds while residues with *brackets* make van der Waals interactions. *F*, superimposition of the *Pf*PNP-MMV000848-PO_4_ ternary complex (this work) and the two binary complexes: *Pf*PNP-inosine (inosine in slate, PDB code: 2BSX) and *Pf*PNP-SO_4_ (PDB code: 1SQ6) (See also Fig. S5) (36). The panel shows as *white sticks Pf*PNP residues surrounding the MMV000848 inhibitor and phosphate ion in the experimental ternary structure (this work).The phosphate and sulfate ions, shown as *orange* and *yellow sticks* respectively, occupy the same location in the active site. Residues involved in the *Pf*PNP–inosine–SO_4_ ternary complex formation (as modeled form the two binary complexes) are colored in slate. MMV, Medicine for Malaria Ventures; *Pf*PNP, *Plasmodium falciparum* purine nucleoside phosphorylase.

To understand the mode of inhibition at the atomic level, we co-crystallized *Pf*PNP with MMV000848 and determined the structure of this complex at 1.85 Å in space group R32 (Fig. 4), which is isomorphous to the structures of *Pf*PNP bound to quinine and mefloquine reported earlier (31). Data collection and refinement statistics are summarized in Table 3. Like in previously reported structures (28, 32, 35, 36), *Pf*PNP forms a homo-hexamer, with three 2-fold symmetrical dimers arranged around a crystallographic three-fold symmetry axis running through the central aperture of the assembly (Fig. 4*A*). The overall structure of the *Pf*PNP monomer (Fig. 4*B* and Video S1) is highly conserved with a r.m.s deviation of 0.30 Å compared to the complexes formed by *Pf*PNP with quinine and 0.15 Å with mefloquine, for a total of 232 superimposed main-chain atoms (18, 37). A conspicuous difference is observed in the active site loop that joins residues Trp212 to Val222 of the enzyme. While this loop is ordered in the complex with transition state analogs such as ImmH where it folds into helix ɑ8, here it is disordered in the complex with MMV000848 (Figs. 4*B* and S3).

**Table 3:** Data collection and refinement statistics of *Pf*PNP-MMV000848

Data collection	*Pf*PNP-MMV000848
Wavelength (Å)	1.000
Space group	R32
Unit cell parameters	a = 95.61 Å, b = 95.61 Å, c = 138.2 Å, ɑ = ß = 90°, ɣ = 120°
Resolution range (Å)^a^	33.17–1.85 (1.916–1.85)
R-merge^b^	0.0743 (0.9243)
I/σ (I)	30.42 (3.73)
Completeness (%)	99.79 (99.32)
Redundancy	20.3 (20.7)
Total reflections	425,760 (42,793)
Unique reflections	20,977 (2065)
Model Refinement	
R-work^c^	0.1736 (0.3293)
R-free^d^	0.1781 (0.3660)
CC_1/2_	1 (0.953)
No. of protein chains	1
No. of protein atoms	1806
No. of ligands	1 (MMV000848)
No. of ligand atoms	23
No. of solvent atoms	170
Wilson B-factor (Å^2^)	27.17
Average B-factor (Å^2^)	39.11
Macromolecules	38.30
Ligands	37.95
Solvent	47.15
R.M.S. deviations	
Bonds (Å)	0.009
Angles (°)	1.14
Ramachandran plot (%)	
Favored region	98.68
Allowed region	1.32
Outlier region	0.00
PDB access code	8W7H

Statistics for the highest-resolution shell are shown in parentheses.

a Statistics for the highest-resolution shell are shown in parentheses.

b R_merge_ = Σ_h_Σ_i_|I_hi_-<I_h_>|/Σ_h,i_ I_hi_, where I_hi_ is the ith observation of the reflection h, while <I_h_> is its mean intensity.

c Rwork = Σ ||F_obs_| − |F_calc_||/Σ |F_obs_|. Calculated over 95% of reflections.

d R_free_ was calculated with 5% of reflections excluded from the whole refinement procedure.

One MMV000848 molecule binds to the active site pocket of each *Pf*PNP monomer, making interactions with residues from both monomers related by crystallographic dyads (having the same color in Fig. 4*B*). A phosphate group is bound in the enzyme active site at a location corresponding to the leaving phosphate ion of the enzyme-inosine product complex reported earlier (Fig. 4, *B*–*E*). The MMV000848 inhibitor (MW = 308.4 Da, 1-carbazol-9-yl-3-cyclopentylamino-propan-2-ol) is composed of one heterocyclic carbazole aromatic group of three fused rings connected to a cyclopentyl-amino five-carbon ring by a propane moiety that comprises a hydroxyl group at position 2 (Fig. 4*E*). The latter forms a hydrogen bond with Asp206, and several water-mediated contacts with Ser91, Asp206 and the hydroxyl group of Tyr160 (Fig. 4, *C*–*E*). The secondary amine of the cyclopentyl-amino group, which is protonated at physiological pH, also forms a water-mediated hydrogen bonds with Asp206 and Ser91 (Fig. 4, *D* and *E*). Thus, the conserved active site residue Asp206 appears key for the stabilization of MMV00848 in the active site pocket. The fused aromatic rings of the carbazole group of MMV000848 fits snugly in the inosine-binding region where it forms an edge-to-face π-π interactions with the Tyr160 phenyl ring and CH-π interactions with Val181 side chain. In addition to these direct contacts, a network of water molecules mediate interactions between the inhibitor and the protein (Fig. 4, *C*–*E*).

Although It does not form direct contacts with MMV000848, the bound phosphate ion plays a key role in stabilizing the complex: it forms hydrogen bonds with Ser91 and Arg88, bifurcated hydrogen bonds with Arg45′ from the other monomer (residues form the monomer partner in the dimer are labeled with a prime) and a water-mediated hydrogen bond with Glu184 (Fig. 4, *C*–*E* and Video S2). Superposition of the structures of *Pf*PNP bound to the inosine substrate (PDB code: 2BSX) and to a sulfate ion (PDB code: 1SQ6) (36) shows a close overlap of the MMV000848 inhibitor with the inosine substrate: the carbazole group occupies the position of the purine ring while the five-carbon ring is located next to the ribose moiety and the phosphate groups completely overlap in the binding pocket (Fig. 4*F*). Only some slight shifts and rotations of side-chains of Tyr160 and His7′ are visible between the two bound structures as seen in Figure 4*F*, revealing an overall structurally well conserved pocket.

### Unfavorable contacts prevents MMV00848 to bind *Hs*PNP

To better understand the structural basis for the observed selectivity of MMV000848 for *Pf*PNP and its lack of binding to the human homolog, MMV000848 was placed into the *Hs*PNP active site using a superimposition of *Pf*PNP with the experimental structures of HsPNP bound to the inosine substrate (38) (PDB code: 1RCT) and to the transition-state inhibitor, ImmH (28, 38) (Fig. S2). *Hs*PNP operates through a “random sequential mechanism” where substrates can bind in any sequence to the enzyme (18, 33, 39, 40). Hence, binding of either the inosine substrate or inhibitor is independent of phosphate-binding. Inosine (18, 38, 41, 42, 43) makes hydrogen bonds with Asn243, Glu201, Tyr88, and His257 of *Hs*PNP, while Met219 and Phe200 form additional interactions with the purine ring of inosine (Fig. S2*A*). Likewise, the *Hs*PNP-ImmH structure (28) reveals a similar set of interactions (Fig. S2*B*). Asn243 initiates the protonation of the N7 atom of the purine ring (18, 33, 39, 40, 42) and Glu201 and Tyr88 are involved in substrate binding (18, 41, 42). An overlay of MMV000848 into either complex revealed that active site residues Asn243 and Glu201 create a polar patch that would provide unfavorable contacts with the hydrophobic carbazole ring of MMV000848. In *Pf*PNP the corresponding patch is formed by hydrophobic residues Val66 of the main chain and Val73 of the neighboring chain, which is essential to accommodate the methyl-thio groups for *Pf*PNP (18, 28, 30, 34, 44). In addition, the side-chain of Tyr88 would sterically clash with the MMV000848 cyclopentyl ring of MMV000848 (Fig. S2, *C* and *D*). Overall, the active site cavity of *Hs*PNP is more hydrophilic and occupies a smaller volume compared to the *Pf*PNP active site which can accommodate the hydrophobic ring of MMV000848. Taken together, these structural features are likely to account for the fine specificity of MMV000848 towards *Pf*PNP and not *Hs*PNP.

To date, most efforts in optimizing the specificity of drugs targeting *Pf*PNP have taken advantage of the dual-specificity of *Pf*PNP for both purines and methylthiopurines (18, 28, 30, 34, 44). This is specific to *Plasmodium* as PNP from other apicomplexa including from *Toxoplasma* (45) are devoid of this dual specificity. Hence, much effort has been given to provide specific interactions with residues next to the 5′ group of the ribose ring (28), specifically, the hydrophobic pocket including Val67, Val 73 and Tyr160 responsible for accommodating the methyl-thio group of immucillin (28, 30, 44). As a result, MT-ImmH is the compound from the immucillin family that discriminates best against *Hs*PNP. Yet, MT-ImmH still binds *Hs*PNP with nanomolar affinity (30) and inhibits *Hs*PNP with a Ki of 0.3 nM (45), which could lead to some toxicity when used for human therapy.

### The case of the active site loop

**Figure 5:**
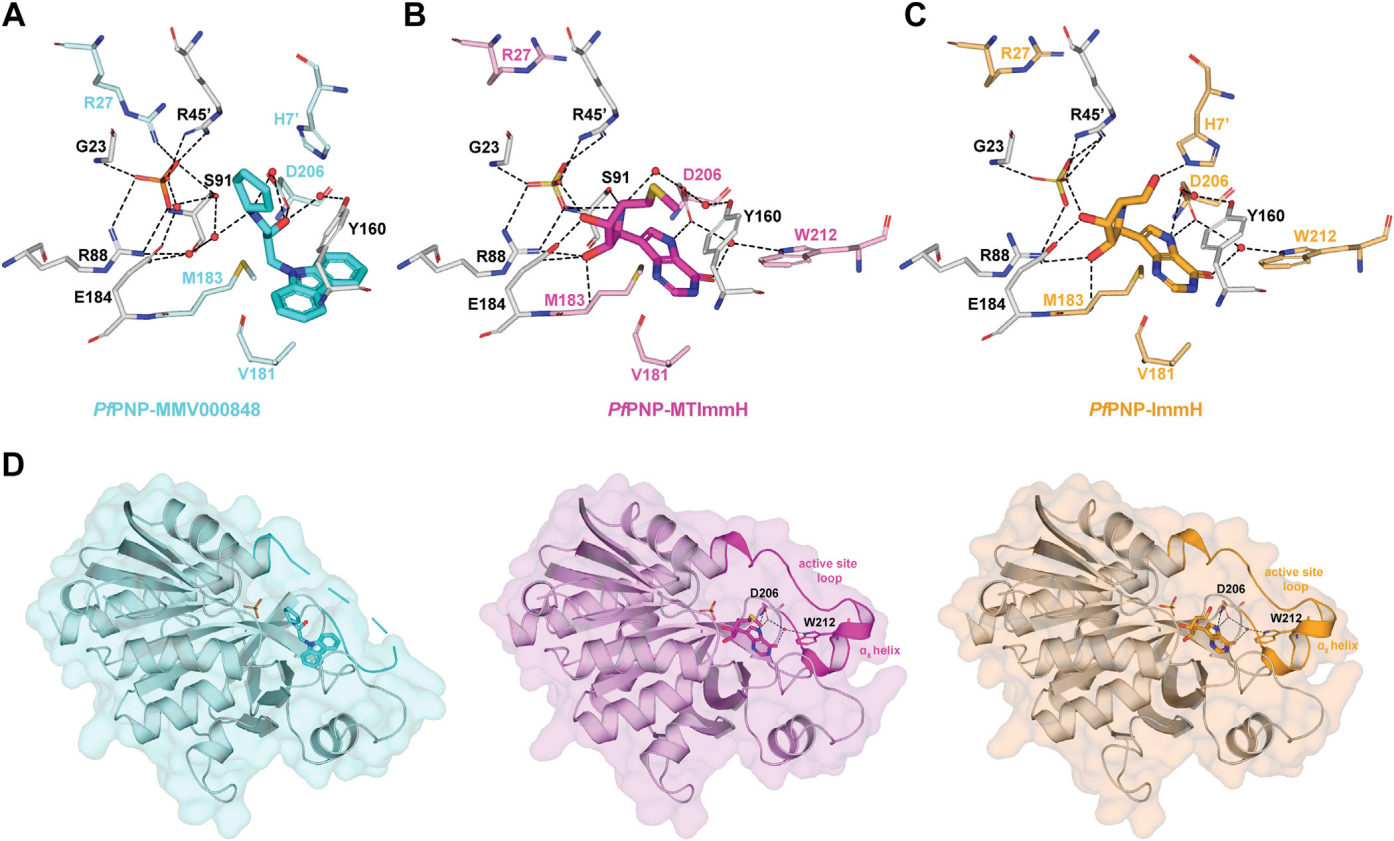
**Flexibility of the active site loop of *Pf*PNP.***A*–*C*, comparison of active site residues involved in binding inhibitor and phosphate between the following complexes: (*A*) *Pf*PNP-MMV000848 (this work), (*B*) *Pf*PNP-MT-ImmH (PDB code:1Q1G) (28), and (*C*) *Pf*PNP-ImmH (PDB code: 1NW4) (28). MMV000848 is shown as sticks in *cyan*, MT-ImmH in *magenta*, and ImmH in *orange*. Active site residues are displayed as *sticks* and colored labels indicate residues whose side-chain adopt various conformations between complexes. *D*, the *Pf*PNP monomer bound with MMV000848 (*left panel*), MT-ImmH (*middle panel*), and ImmH (*right panel*) shown as a transparent surface with the active site loop labeled. The active site loop is ordered in both the *Pf*PNP–MTImmH and *Pf*PNP–ImmH complexes, with the formation of helix ɑ_8_. In particular, residue Trp212 of the active site loop and Asp206 are involved in water-mediated interactions with the ligands. Conversely, in the *Pf*PNP–MMV000848 complex, the corresponding segment is disordered (residues 212–221 dashes). MMV, Medicine for Malaria Ventures; *Pf*PNP, *Plasmodium falciparum* purine nucleoside phosphorylase.

In contrast to MMV000848 that specifically binds and inhibits *Pf*PNP but not the human ortholog, inhibitors of the immucillin family, such as ImmH and MT-ImmH strongly inhibit both *Hs*PNP and *Pf*PNP with K_i_ values of 0.6 nM and 2.7 nM respectively (22, 29, 45). An alignment of the amino-acid sequences of *Hs*PNP and *Pf*PNP is displayed in Fig. S4. Due to the low amino-acid sequence identity of 9% between *Pf*PNP and *Hs*PNP, a structure-based alignment (46) was performed, revealing a r.m.s deviation of 2.3 Å over 193 Cɑ atoms. To understand the origin of high-affinity binding and broad reactivity, we examined the structures of *Pf*PNP bound with MT-ImmH (PDB code: 1Q1G) (28) and with ImmH-SO_4_ (PDB code: 1NW4) (28) (Fig. 5). While the active site loop spanning residues 207 to 225 (28, 29, 45) of *Pf*PNP-MMV000848 is flexible, the corresponding residues in both immucillin complex structures are ordered (Figs. 5 and S3). As a result, both immucillin inhibitors form water-mediated hydrogen bonds with Trp212 from the active site loop, while these interactions are not observed with MMV000848 due to disorder of the corresponding residue (Fig. 5). Moreover, compared to the *Pf*PNP-MMV000848 complex, Asp206 is shifted by 1.5 Å deeper into the substrate-binding cavity enabling it to directly form a hydrogen bond with the N7 atom of the purine ring of ImmH and MT-ImmH. In contrast, Asp206 only forms a weaker water-mediated hydrogen bond with the hydroxyl group of MMV000848. Likewise His7′ forms a hydrogen bond with the 5′ hydroxyl group of ImmH but does not interact with MMV000848. (Fig. 5, *A*–*C*). Thus, additional hydrogen bonds with the immucillin in part due to the ordering of the active site loop to cover the inhibitor, are likely to explain the higher affinity of *Pf*PNP for both immucillin compounds with respective K_D_ values of 2.7 nM and 860 PM respectively (45) compared with MMV000848. Examination of crystal lattice contacts in the vicinity of the active site loop revealed that its conformation was unlikely to be affected by crystal packing forces. The active site loop undergoes a disorder-order transition and is stabilized only in the transition state as shown for PNP from various species (18, 35, 47, 48). Hence, despite sharing an overlapping binding site in the *Pf*PNP catalytic center with immucillin compounds, MMV000848 appears to use a different mode of action for inhibiting enzyme activity: instead of acting as an inhibitor of transition-state formation MMV000848 rather stabilizes a pre-transition state of the enzyme. This agrees with the observed conformation of Arg27, a residue that stabilizes the negative charge formed on the phosphate during this intermediate state (35, 42, 47, 49) as seen in the complex with MMV000848 and also in the *Pf*PNP bound to inosine and sulfate (Fig. S5),while Arg27 points away from the phosphate group in the immucillin-bound complexes (Fig. 5, *A*–*C*).

### Comparison with quinolines inhibitors of *Pf*PNP

**Figure 6:**
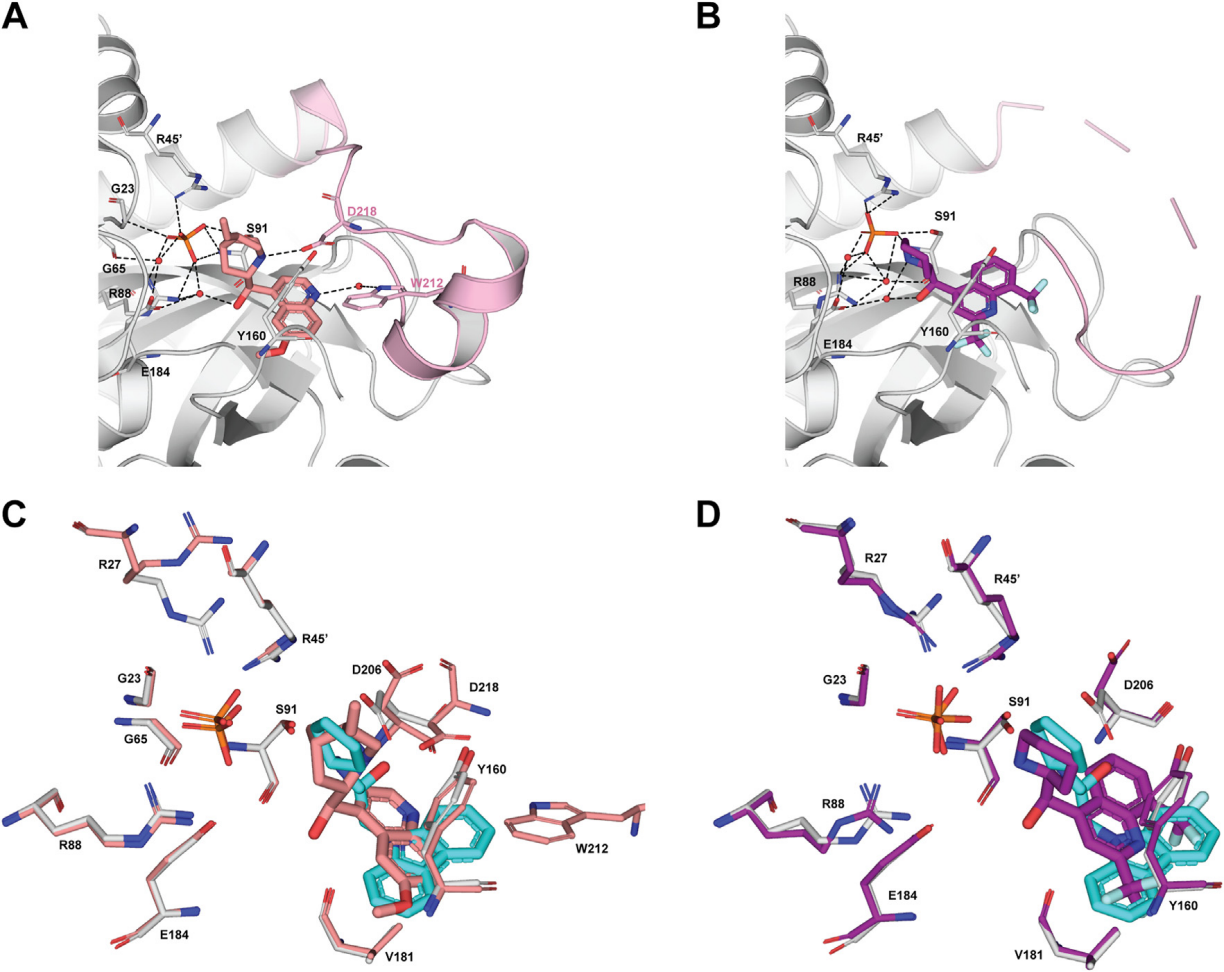
**Comparisons with quinoline inhibitors.***A*, *Pf*PNP–quinine complex (PDB code: 5ZNC) (31). Quinine is shown as *salmon sticks* and the phosphate group as *orange sticks*. Quinine forms hydrogen bond interactions with Trp212 (water-mediated) and Asp218 of the active site loop, as well as with Glu184. *B*, view of the *Pf*PNP–mefloquine complex (PDB code: 5ZNI) (31). Mefloquine is shown as *purple sticks* and the phosphate ion as *orange sticks*. Mefloquine forms a single water-mediated hydrogen bond with Glu184. For (*A* and *B*), residues interacting with inhibitors are shown in *sticks* and the active site loop is colored in *light pink* with *dashes* representing missing atoms. Hydrogen bonds are depicted as *black dashes* and water molecules involved in binding as *red spheres*. *C*, superposition of the active site of *Pf*PNP-MMV000848 (*white sticks*) with *Pf*PNP-quinine (*salmon sticks*). *D*, superposition of the active site of *Pf*PNP–MMV000848 (*white sticks*) with the *Pf*PNP–Mefloquine complex (*purple sticks*). In panels (*C* and *D*), MMV000848 is shown as *cyan sticks* in the same orientation. MMV, Medicine for Malaria Ventures; *Pf*PNP, *Plasmodium falciparum* purine nucleoside phosphorylase.

Using a similar CETSA based approach as employed here, quinine and mefloquine were previously found to bind and inhibit *Pf*PNP (31). A comparison of these two bound structures (PDB codes: 5ZNC and 5ZNI respectively) with the *Pf*PNP-MMV000848 complex structure (this work) is given in Figure 6. A conserved feature is the formation of edge-to-face π-stacking interactions with the fused aromatic rings of each of the three compounds with Tyr160; *e.g.*: with the quinoline rings for quinine and mefloquine and the carbazole ring for MMV000848 (Fig. 6). While for all three structures, interactions with the phosphate ion remain largely conserved, the hypoxanthine-binding region of the active site reveals some differences: quinine makes additional hydrogen-bond interactions with Trp212 and Asp218, that project from the active site loop. These interactions are accompanied with an ordering of the active-site loop in the complex with quinine while the loop is flexible in the complex with mefloquine (Fig. 6*B*) and with MMV000848 (Fig. 5*D*). Thus, loop stabilization *via* interactions with quinine is likely to account for the stronger inhibition observed with this drug. Overall, these structural features agree with the ranking in inhibition potency and binding affinity of the three inhibitors: quinine> MMV000848> mefloquine and also correlates with a higher number of hydrogen bonds stabilizing the complexes with quinine and MMV000848 compared to the complex with mefloquine (Table S2). We also note variability in the active site loop whose conformation differs between *Pf*PNP-quinine and transition state complexes (28, 29, 35). Hence, stabilization of the substrate loop appears crucial for the design of potent active site inhibitors. Our *in vitro* data also indicated that neither quinoline compounds inhibits *Hs*PNP (Fig. 3*A*). This derives from the presence of the aromatic quinoline rings in both quinine and mefloquine, which would provoke steric clashes with *Hs*PNP active site residues. Moreover, the presence of an aromatic ring moiety close to the active site loop appears crucial for designing *Pf*PNP inhibitors that are able to discriminate against *Hs*PNP.

### Isobologram analysis suggests antagonistic effect between MMV000848 and PfPNP inhibitors

**Figure 7:**
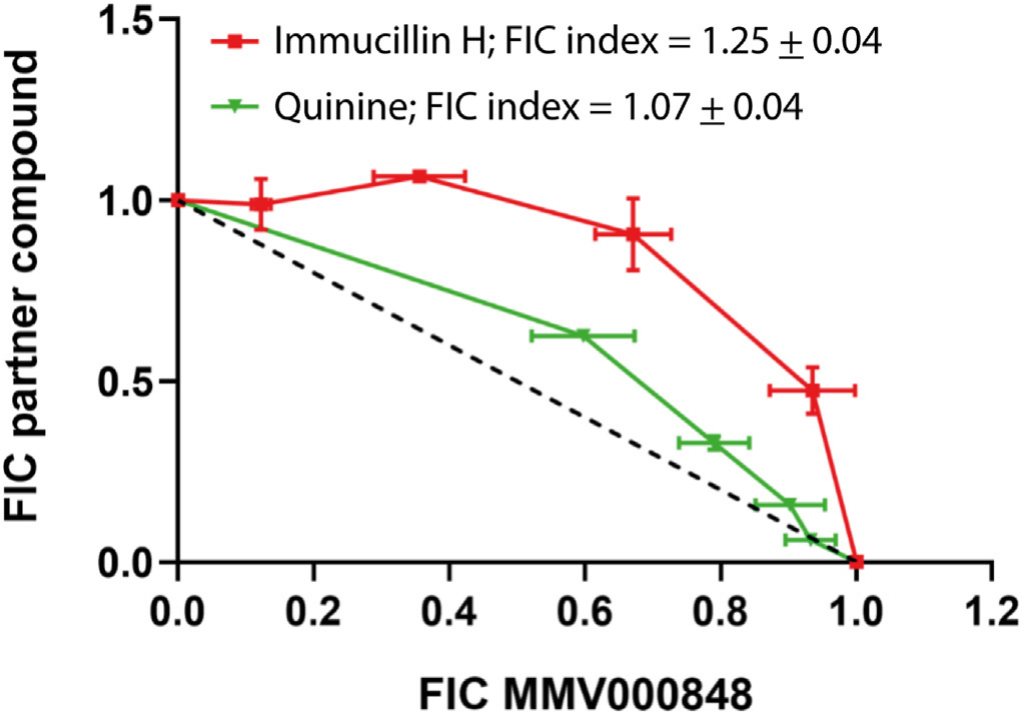
**Isobologram analysis.** Synergy analysis for MMV000848 in combination with either Immucillin H or quinine against the WT 3D7 parasite (“3D7 WT”). Isoboles representing fractional inhibitory concentration (FIC) of each drug in combination are plotted across a range of drug pair concentrations. FIC indexes and their respective SDs represent the average of all FICs in each strain. *Dotted* line represents reference isobole indicating Loewe additivity model. MMV, Medicine for Malaria Ventures.

Taken together, MS-CETSA, biophysical and structural data point to *Pf*PNP as a possible target for MMV000848. To investigate this proposed mechanism of action to kill the parasite at the intra-erythrocyte stage, we tested whether any synergistic or antagonistic effect could be detected by pairing MMV000848 with known inhibitors of *Pf*PNP enzymatic activity that can also kill the parasite. ImmH was previously shown to kill the parasite *via* a direct competition with the inosine substrate in binding *Pf*PNP; importantly, ImmH also targets *Hs*PNP (22, 28, 29, 30). The second drug tested was quinine which inhibits *Pf*PNP but not *Hs*PNP (Fig. 3*A*). For both pairs tested MMV000848/ImmH and MMV000848/quinine, a subadditivity effect was found in killing the *falciparum* 3D7 strain, although the antagonistic effect observed was weaker for the MMV000848/quinine pair (Fig. 7). Competition between MMV000848 and either quinine or ImmH for the same binding site on *Pf*PNP is likely the cause of the observed antagonistic effect in this cell assay. As for the origin of the weaker subadditivity effect, it should be borne in mind that *Pf*PNP inhibition constitutes only part of the proposed mechanism of action of quinine and other mechanisms such as inhibition of heme polymerization and heme catalase activity have been demonstrated (50).

## Discussion

Despite decades of research efforts and the availability of several frontline prophylactic and chemotherapeutic treatments, malaria remains a major health problem with 247 million recorded cases in 2021 according to WHO (https://www.who.int/publications/i/item/9789240064898). The situation is aggravated by the emergence of parasites resistant to artemisinin based combination therapies that include -in addition to dihydro-artemisinin- a longer half-life partner drug such as lumefantrine, piperaquine or mefloquine. Thus, in order to combat malaria and the rise of resistant parasites, a better understanding of the mode(s) of action of existing drugs and drug candidates is likely to help the development of novel potent compounds. As part of this effort, we embarked in a systematic drug deconvolution program of known drugs such as quinolines and MK-4815 (a candidate compound from Merck (51)) and also on drug candidates such as those included in the MMV “malaria box” (10).

Quinine, a natural alkaloid initially isolated from the bark of cinchona trees, was historically extremely successful until resistance occurred leading to the development of several analogs. Remarkably, using an agnostic MS-CETSA method, *Pf*PNP was proposed to constitute a possible target accounting for part of quinine mode of action (31). In addition to a protein thermal stabilization effect provided by quinine in a proteomics assay, evidence in favor of *Pf*PNP as a target of quinine included a submicromolar dissociation constant, a competitive mode of inhibition with respect to the inosine substrate as well as structural evidence provided by X-ray crystallography showing quinine bound at the *Pf*PNP active site (31).

However, as exemplified by the complex case of falcilysin which has a promiscuous pocket in its large active site chamber, binding and even enzyme inhibition provoked by binding does not necessarily imply a unique mode of action (32). Thus, it is possible that the mechanisms of action employed by several antimalarial drugs are pleiotropic *i.e.*: that these compounds work *via* hitting several targets in the parasite. Indeed, both knockdown and drug sensitivity assays point in the direction of a complex mode of action for many compounds and do not favor the simple paradigm of “one drug hitting one target”. In the case of MMV000848, a 3-fold difference was observed between its dissociation constant with *Pf*PNP (K_D_ = 1.52 μM) and its anti-parasitic activity (EC_50_ = 0.5 μM). When considering the higher enzymatic IC50 value as well, this indicates that *Pf*PNP inhibition might account for only part of the mechanism of action of MMV000848 in killing the parasite. Indeed, the CETSA results indicate that MMV000848 can also interact with several other malarial proteins besides *Pf*PNP (Tables 1 and 2) and the combined effects of several interactions could lead to the observed anti-parasitic EC50. Moreover, we cannot formally exclude that some interactions made by MMV000848 not detected by CETSA could also contribute to the mode of action of this drug candidate.

Nonetheless, even if not constitutive of a definitive proof of mode of action, the MS-CETSA method employed here to deconvolute possible targets for MMV00848 leads to identification of *Pf*PNP as a prime candidate. In the present work, using MS-CETSA screening approach we were able to identify putative protein targets of MMV000848, a malaria box compound, which has demonstrated activity against asexual-stage *P. falciparum.* (10) We found that in the presence of MMV000848, *Pf*PNP exhibited a very strong increase in its melting temperature. Moreover, further enzymatic, biophysical and structural characterization demonstrated a competitive mode of inhibition and binding to the active site of the plasmodium enzyme was unambiguously shown by X-ray crystallography, disclosing a mode of interaction that can now be improved using medicinal chemistry informed by a high resolution structure. Interestingly, in contrast to the immucillin family of inhibitors (29, 30), the human enzyme is not affected by MMV000848 which suggests that specific inhibition of the parasite enzyme is possible, which is advantageous from a toxicity perspective (28, 29, 30).

Structural comparison of MMV000848 with quinine and mefloquine shed light on a common pattern of interactions established between the aromatic ring and Tyr160. While the bulky aromatic rings of MMV000848, quinine and mefloquine are unable to fit in the active site of *Hs*PNP, they are key in maintaining hydrophobic interactions with Tyr160 of *Pf*PNP. Interestingly, while the corresponding loop is disordered in the *Plasmodium* enzyme bound to MMV000848, hydrophobic residues Pro209, Trp212 and Phe217 located on the active site loop forms significant π-stacking and van der Waals interactions with the hypoxanthine substrate (35). Thus, further drug design based on MMV000848 should aim at establishing additional interactions with residues projecting from the active site loop which is likely to yield more potent inhibitors against *Pf*PNP while retaining specificity for the *Plasmodium* enzyme. Another possible avenue for improving inhibitor potency is to attempt to grow the molecule to reach into the phosphate-binding site which is systematically found in ternary complexes formed between the enzyme with either an inhibitor or substrate respectively. Compared to previously reported *Pf*PNP inhibitors (Fig. S6) which all contain an aromatic 2-ring system (pyrrolopyrimidinone for ImmH and MT-ImmH; quinoline for mefloquine and quinine), MMV000848 contains an aromatic 3-ring system. This feature should facilitate the design of antimalarials by expanding the available choices of scaffolds for the rational design of *Pf*PNP inhibitors.

## Experimental procedures

### Parasite culture

*P. falciparum* 3D7 cultures were maintained in Malaria Culture Media (MCM) containing RPMI-1640 medium (Gibco) supplemented with 0.25% Albumax II (Gibco), 0.1 mM hypoxanthine (Sigma), 0.2% sodium bicarbonate (Sigma), and 10 mg/l gentamycin. Human blood group O were used for parasite culture and cultures were maintained at 37 °C with 5% CO_2_, 3% O_2_, and 92% N_2_. Culture media were replenished daily with freshly packed RBC added to the culture when necessary.

### Sample preparation for ITDR MS-CETSA and LC/MS analysis

Samples for intact-cell and lysate ITDR MS-CETSA and LC/MS analysis were prepared as described in Dziekan *et al.* (13) For both intact-cell and lysate MS-CETSA, synchronized mid-trophozoite stage (28 + 4 HPI) culture at 10% parasitemia and 2% hematocrit was used. In case of intact-cell MS-CETSA, 1 ml of packed blood was loaded on MACS CS column (Milteny Biotech), washed, and eluted. Enriched infected RBC (>80% parasitemia) were then incubated for 1 h in MCM at 37 °C with agitation for recovery and equilibration. The infected RBC were then divided into ten aliquots (75–90 million cells in 6 ml MCM for each aliquot) and treated with 4-fold dilution series of MMV000848 at 10 μM top concentration or an equivalent volume of their respective solvent at 0.1% concentration, and incubated for 1 h at 37 °C with agitation. Cells were pelleted by centrifugation at 2500 rpm for 5 min, washed with PBS, resuspended in 150 μl PBS per aliquot, and transferred in 50 μl aliquots to three 96-well plates, each representing different heat challenge temperature (37 °C, 50 °C, or 60 °C). Samples were subjected to thermal challenge for 3 min, followed by 3 min cooling at 4 °C. After heat treatment, 50 μl of lysis buffer (50 mM Hepes pH 7.5, 5 mM ß-glycerophosphate, 0.1 mM Na_3_VO_4_, 10 mM MgCl_2_, 2 mM tris(2-carboxyethyl)phosphine) (TCEP), and cocktail EDTA-free protease inhibitors (Sigma)) were added to each well. Samples were then subjected to three times of freeze/thaw cycle, followed by mechanical shearing and centrifugation at 20,000*g* for 20 min at 4 °C to isolate the soluble protein fraction. Protein quantification was then performed with bicinchoninic acid protein assay kit (Pierce). In case of lysate MS-CETSA, the parasite culture was pelleted and incubated with 10× volume of 0.1% saponin in PBS (pH 7.2) for 5 min. Following centrifugation at 2500*g* for 5 min, the supernatant containing RBC cytosol was removed, while intact parasite pellet was washed three times with 50 ml of ice-cold PBS. Parasite was then resuspended in 1 ml of lysis buffer and subjected to three times of freeze/thaw cycle, followed by mechanical shearing. Parasite lysate was then centrifuged at 20,000*g* for 20 min at 4 °C and supernatant containing soluble parasite proteins was isolated. Protein concentration was quantified with bicinchoninic acid protein assay kit. Ten aliquots of 20 μg of proteins were added to MMV000848 100 μM top concentration or an equivalent volume of their respective solvent at 0.1% concentration, incubated at room temperature for 3 min, and heated (37 °C, 50 °C, 55 °C, or 60 °C) for 3 min followed by 3 min cooling at 4 °C. The post-heating lysates were centrifuged at 20,000*g* for 20 min at 4 °C and the supernatant was transferred to a fresh Eppendorf tube for further processing.

### Peptide preparation and labeling

Following quantification of lysate and intact-cell samples, the volume equivalent to 20 μg total protein in post-heating treatment was aliquoted and incubated with denaturation and reduction buffer (100 mM triethylammonium bicarbonate (pH 8.5), 20 mM TCEP (pH 7.0), and 1% (w/v) Rapigest (Waters)) at 55 °C for 20 min, followed by alkylation with 55 mM chloroacetamide at room temperature for 30 min. Samples were then sequentially digested with Lys-C (0.23 AU LysC/μg of protein, Wako) for 3 h, followed by trypsin (0.05 μg trypsin/μg of protein) for 18 h at 37 °C. Enzyme activation and Rapigest degradation was performed by incubation with 1% TFA (Sigma) for 45 min at 37 °C and the sample supernatant containing peptides was collected by centrifugation at 20,000g for 10 min. The samples were then dried in a centrifugal vacuum evaporator and resolubilized with 200 mM triethylammonium bicarbonate to 1 μg/μl concentration. Peptides were labeled with TMT10plex Isobaric Label Reagent Set (Thermo Fisher Scientific) according to manufacturer’s protocol. Briefly, 4 μg of the digested protein was labeled for at least 1 h at a condition of pH >6 and then quenched with 1 M Tris, pH 7.4. The labeled samples were subsequently pooled and desalted using an Oasis HLB column (Waters), followed by vacuum drying. Samples were resuspended in 5% (v/v) acetonitrile, 5% (v/v) ammonia and separated using high pH reverse phase Zorbax 300 extend C-18 4.6 mm × 250 mm (Agilent) column and 1260 Infinity II LC system (Agilent) was used for offline sample pre-fractionation into 96 fractions. The fractions were concatenated into 20 fractions and dried with a centrifugal vacuum concentrator.

### LC/MS analysis

The dried peptide sample fractions were resuspended in 1% acetonitrile, 0.5% (v/v) acetic acid, and 0.06% TFA in water prior to analysis on LC/MS. Online chromatography was performed using the reverse phase liquid chromatography Dionex 3000 UHPLC system coupled to a Q Exactive HF mass spectrometer (Thermo Fisher Scientific). Each fraction was separated on a 50 cm × 75 μm Easy-Spray analytical column (Thermo Fisher Scientific) in 70 min gradient of programmed mixture of mobile phase A (0.1% formic acid) and mobile phase B (99.9% acetonitrile, 0.1% formic acid) to the following gradient over time: 1 to 55 min (2–25%), 55 to 57 min (25–50%), 57 to 58 min (50–85%), 58 to 63 min (85%), 63 to 70 min (2%). MS data were acquired using data-dependent acquisition with full scan MS spectra acquired in the range of 350 to 1550 m/z at a resolution of 70,000 and AGC target of 3e6; Top12 MS2 35,000 and AGC target of 1e5, and 1e5 isolation window at 1.2 m/z.

### *Pf*PNP expression and purification

*Pf*PNP was expressed and purified as previously described (36). Briefly, *Pf*PNP gene was subcloned into pNIC-CH vector and expressed in Rosetta BL21-DE3 *Escherichia coli* in LB broth supplied with 50 μg/ml kanamycin and 34 μg/ml chloramphenicol. Cells were grown at 37 °C until an OD_600_ of 0.6 to 0.8 was reached and expression was induced with 0.8 mM IPTG at 16 °C overnight. Cells were harvested by centrifugation at 4000*g* for 20 min at 4 °C. The cell pellet was resuspended in lysis buffer (20 mM Hepes, 500 mM NaCl, 10% (v/v) glycerol and 1 mM TCEP, pH 7.5) and lysed *via* sonication on ice at 55% amplitude, 5 s ON/OFF for 5 min. Lysate was clarified by centrifugation at 47,000*g* for 45 min at 4 °C, and the supernatant was filtered using a 0.22 μM filter to remove cell debris. Filtered supernatant was purified using a 2-step purification process of immobilized metal affinity chromatography (IMAC) followed by size exclusion chromatography (SEC). The supernatant was first loaded on a pre-equilibrated HisTrapHP column (GE Healthcare) and washed with IMAC wash buffer 1 (20 mM Hepes, 500 mM NaCl, 15 mM imidazole, 10% (v/v) glycerol and 1 mM TCEP, pH 7.5) and IMAC wash buffer 2 (20 mM Hepes, 500 mM NaCl, 30 mM imidazole, 10% (v/v) glycerol, and 1 mM TCEP, pH 7.5). Bound protein was eluted with a gradient from 30 mM imidazole to 500 mM imidazole. The protein was then concentrated and loaded onto a HiLoad 16/60 Superdex 200 column (GE Healthcare) pre-equilibrated with SEC buffer (50 mM KH_2_PO_4_, 10% (v/v) glycerol, 1 mM TCEP, pH 7.4). Fractions containing the final purified protein were confirmed using SDS-PAGE, pooled, and concentrated.

### *In vitro* thermal shift assay

Initial confirmation of *in vitro* stabilization of *Pf*PNP by MMV000848 was determined using the thermal shift assay in SEC buffer. In a 25 μl reaction, 15 μM *Pf*PNP protein was incubated with 5X SYPRO orange dye and various concentrations of MMV000848, chloroquine, or ImmH (ranging from 100 μM to 1.5625 μM). RFU was measured in triplicates using the CFX96 Touch Real-Time PCR Detection System (Bio-Rad) on the FRET channel from 25 °C to 95 °C. Raw RFU values were exported and analyzed using Graphpad Prism to determine the melting temperatures (T_m_) of *Pf*PNP in the presence of MMV000848.

### Isothermal titration calorimetry

ITC experiments were carried out in using the MicroCal PEAQ-ITC system (Malvern Panalytical). The cell contained *Pf*PNP or *Hs*PNp and the titration syringe contained the compound solution. Both cell and syringe contents were diluted in SEC buffer with the same DMSO concentration. Titrations were initiated with a single 0.4 μl injection followed by 18 2 μl injections at 25 °C in a single ITC experiment. Stirring speed was kept at 750 rpm. The heat peaks integration and nonlinear regression analysis were carried out using MicroCal PEAQ-ITC analysis software (Malvern Panalytical) and fitted using the One Set of Sites fitted offset model to determine the K_D_, N (sites) and ΔH.

### *Pf*PNP enzymatic activity assay

The *Pf*PNP enzymatic activity assay (36) was used to determine the IC_50_ of MMV000848 and quinoline compounds as well as the enzyme kinetics of MMV000848. Experiments were carried out in triplicates in a reaction buffer of 50 mM potassium phosphate pH 7.5.

For determination of IC_50_, each 100 μl reaction mixture contained 60 mU/ml xanthine oxidase (Sigma) and 200 μM inosine (Sigma) in the presence and absence of varying concentrations of inhibitors quinine, mefloquine and MMV000848 (1 mM – 488.28 nM), and ImmH (10 μM–4.88 nM for *Hs*PNP and 200 μM–97.66 nM for *Pf*PNP). Reactions were initiated with the automated addition of 100 nM *Pf*PNP and followed for a total duration of 1 min. TECAN 10 M plate reader was used to monitor the coupled reaction by measuring absorbance at OD_293nm_. The initial velocity was calculated automatically using Magellan software provided by the manufacturer. The V_mean_ values obtained were plotted against the log of the inhibitor concentration in GraphPad Prism to obtain the IC_50_ values.

For determination of enzyme kinetics, a similar set-up was used whereby each 100 μl reaction mixture contained 60 mU/ml xanthine oxidase but in the presence of varying concentrations of inosine (500 μM–7.8125 μM) and MMV000848 (50 μM–1.5625 μM). V_mean_ values obtained were plotted against substrate concentration in GraphPad Prism and fit using the Michaelis Menten model to obtain the V_max_ and apparent K_M,app_ of *Pf*PNP for inosine in the presence of different concentrations of MMV000848. V_max_ values in μmol • mg^−1^ • min^−1^ were obtained by converting ΔOD_293nm_ measured using the Beer-Lambert law and taking into account the extinction coefficient of uric acid (12.9 mM^1^ • cm^−1^). To obtain K_i_, the same values were fit to the competitive inhibition model in GraphPad Prism. Measurements were performed on multiple 96-well plates. To account for plate-to-plate variation, a control, which contains 100 nM *Pf*PNP, 60 mU/ml xanthine oxidase, 500 μM inosine, and 1% DMSO, was included in each plate, representing 100% velocity. All velocities were normalized to the control on the same plate. Lineweaver-Burk graph was obtained by plotting reciprocal of normalized velocities against reciprocal of inosine concentrations. Error bars denote SD in all graphs.

### Crystallization, data collection, and processing

The *Pf*PNP-MMV000848 complex structure was obtained by incubating 37 mg/ml of *Pf*PNP with 3 mM of MMV000848 on ice for 15 min followed by screening for crystallization conditions of co-crystals with commercial screening kits (JCSG-plus and Morpheus from Molecular Dimensions, PEG/Ion Screen from Hampton Research). Co-crystal screens were set up with a mosquito crystallization robot (TTP Labtech) and sitting-drop vapor diffusion method at 20 °C. Multiple crystal hits were observed after 5 days and left to grow up to 14 days. Crystals were fished in nylon loops and flash-frozen in liquid nitrogen for data collection. The complex structure of *Pf*PNP and MMV000848 was obtained from co-crystals grown in buffer condition of JCSG+ C12 (10% (w/v) PEG 1000).

X-ray diffraction intensities were collected at the ANSTO MXI beamline, integrated, and scaled using XDS (52). Structures were solved with molecular replacement as implemented in the program Phaser (53) using the *P. falciparum* ligand-free structure (PDB access code: 5ZNI (31)) as a search probe. Refinement was done using the Phenix (54) package and interspersed with manual model correction using Coot (55).

### Isobologram analysis

For drug interaction study, normal MCM was replaced with MCM without hypoxanthine supplementation (purine-free media). Prior to the start of assay, ring stage parasites (∼4–10 h post-infection) were washed with PBS for three times to remove residual hypoxanthine from the culture. IC_50_ measurements were conducted for each drug alone and for combinations at fixed volumetric ratios (*i.e.*, immucillin H or quinine: MMV000848 ratio - 5:0, 4:1, 3:2, 2:3, 1:4, 0:5). Parasites were incubated for 48 h at 37 °C. After incubation, the number of new, viable parasites in each well on the subsequent replication cycle was quantified by flow cytometry. Cells were stained using 50 μl of 8 μM Hoechst 33,342 in PBS (pH 7.2) for 15 min at 37 °C, followed by addition of ice-cold 200 μl of PBS. Cells were quantified using LSR Fortessa X-20 Flow Cytometer (BD Biosciences) using UV laser (355 nm) and results were analyzed with FACS Diva Software (BD Biosciences). The assay was performed as biological triplicates. The isobologram analysis was conducted according to the Loewe additivity model, in which the average sum of fractional inhibitory concentrations (FIC index) for the two inhibitors in a combination is indicative of additivity, synergism, or antagonism. The combination with FIC index greater than 1 is considered antagonistic, less than 1 synergistic and if equal to 1 additive (56).

## Data availability

Atomic coordinates and structure factors for the *Pf*PNP-MMV000848 complex reported here have been deposited with the PDB (www.rcsb.org) with access code 8W7H.

## Supporting information

This article contains supporting information.

## Conflict of interest

The authors declare that they have no conflicts of interest with the contents of this article.
