# Supplementary material for: Identification and structural validation of purine nucleoside phosphorylase from Plasmodium falciparum as a target of MMV000848
Source: J Biol Chem. 2023 Dec 21;300(1):105586. doi: 10.1016/j.jbc.2023.105586 (PMC10911062; doi:10.1016/j.jbc.2023.105586)
Supplement: Supporting information [file mmc3.docx]

**Identification and structural validation of purine nucleoside phosphorylase from *Plasmodium falciparum* as a target of MMV00848.**

Zara Chung^1,2,#^, Jianqing Lin^1,2,#^, Grennady Wirjanata^1^, Jerzy M. Dziekan^1,4^, Abbas El Sahili^1,2^, Peter R. Preiser^1,3^, Zbynek Bozdech^1,2,*^ & Julien Lescar^1,2,3,*^,

**Supplementary information**

6 Figures, 2 Tables, 2 videos

**
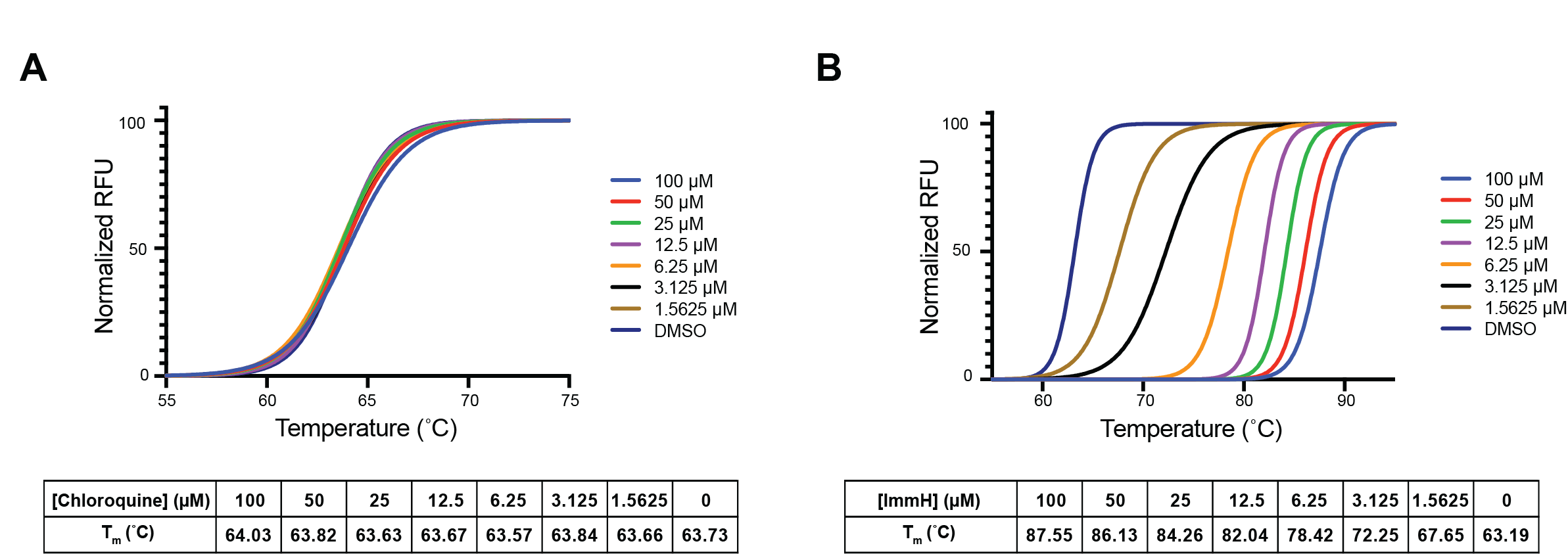
**

**Supplementary Figure 1. Normalized melting curve from Differential Scanning Fluorimetry (DSF) of *Pf*PNP stabilization by chloroquine and ImmH.** DSF was performed with 15 µM of *Pf*PNP and various concentrations (100 µM to 1.25625 µM) of chloroquine (**A**) or ImmH (**B**). Chloroquine was used as a negative control as it does not bind nor inhibit *Pf*PNP. Accordingly chloroquine does not induce *Pf*PNP stabilisation as measured via DSF. ImmH shows strong binding and stabilisation of *Pf*PNP with increasing drug concentration and was used as positive control.


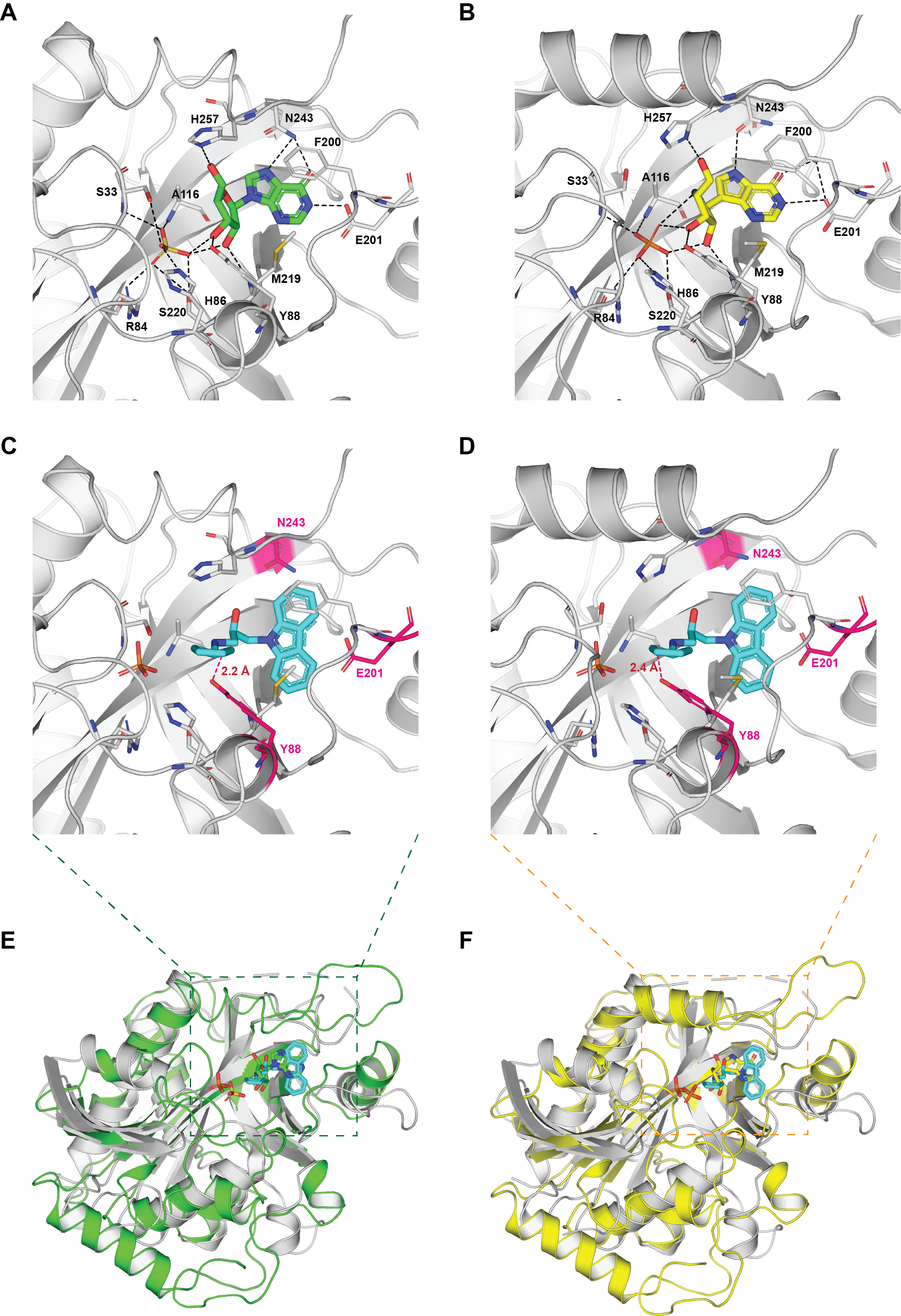


**Supplementary Figure 2. Structural overlay indicates unfavourable contacts between MMV000848 and residues from the *Hs*PNP active site. A.** View of the experimental *Hs*PNP-inosine complex structure with inosine (green sticks) and the sulfate ion (yellow sticks) interacting with active site residues (PDB code: 1RCT). **B.** Experimental structure of *Hs*PNP bound with ImmH (yellow sticks) and phosphate (orange sticks) (PDB code: 1RR6). Hydrogen bonds between the ligands and active site residues of *Hs*PNP are shown as dashes. **C and D.** Overlays of the *Pf*PNP-MMV000848 experimental complex (this work) with *Hs*PNP complex structures depicted in panels A and B. MMV000848 is displayed as cyan sticks at the active site of *Hs*PNP (as bound with inosine) (panel **C**) or at the active site of *Hs*PNP (as bound with ImmH) (panel **D**). Following protein superimposition, the *Pf*PNP, inosine and ImmH molecules were occluded for clarity. Charged and polar catalytic residues Asn243, Glu201 and Tyr88 that would provide unfavourable contacts with MMV000848 are coloured in pink. The close distance between Tyr88 and the cyclopentyl group of MMV000848 is shown as red dashes. **E.** Overall view of the superposition of *Pf*PNP (grey ribbons) bound to MMV000848 (cyan sticks) with *Hs*PNP (green ribbons) bound to inosine (green sticks). After superimposition, the overall r.m.s. deviation between both PNP structures is 2.34 Å for 193 equivalent α-carbon atoms. **(F).** Overall view of the superposition of *Pf*PNP (grey ribbons) bound to MMV000848 (cyan sticks) with *Hs*PNP (yellow ribbons) bound to ImmH (yellow sticks). After superimposition, the overall r.m.s. deviation between both structures is 2.31 Å for 193 equivalent α-carbon atoms.

**
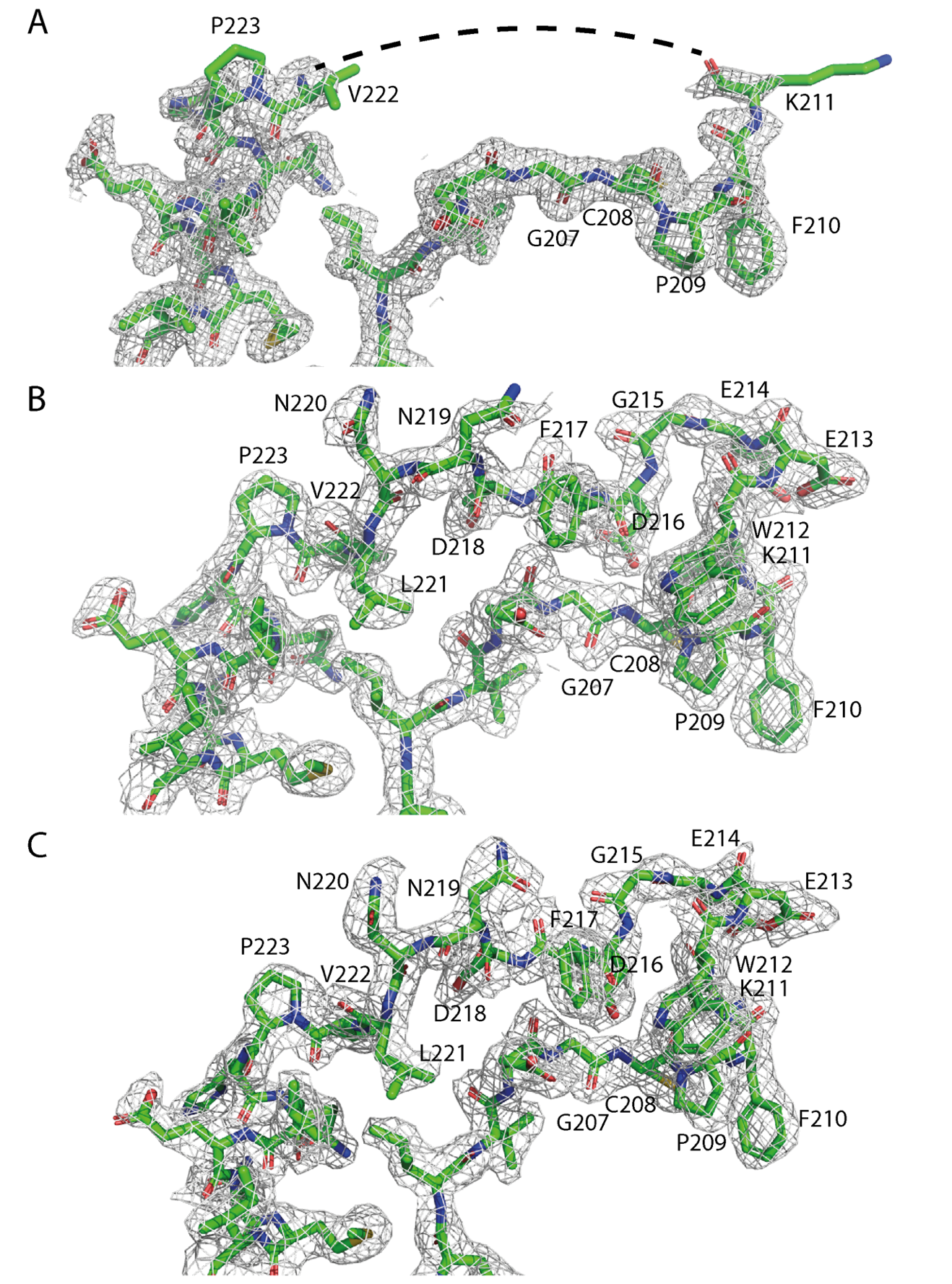
**

**Supplementary Figure 3. Comparison of difference Fourier electron density maps in the region containing the active site loop of *Pf*PNP.** Electron density Fourier maps with 2F_0_-F_c_ amplitude coefficients and phases from the refined models are shown for the following complexes (**A**) *Pf*PNP-MMV000848 (this work) (**B**) *Pf*PNP-MT-ImmH (PDB code: 1Q1G), (**C**) *Pf*PNP-ImmH (PDB code: 1NW4). Amino-acid residues of *Pf*PNP are shown as green sticks and 2F_0_-F_c_ electron density maps are shown at a level of 1.0 σ above the mean electron density value. Clear electron density is visible for the active site loop in both the *Pf*PNP-MT-ImmH and *Pf*PNP-ImmH complexes, but not in the *Pf*PNP-MMV000848 complex where residues 212-221 are flexible (indicated by a dashed line).


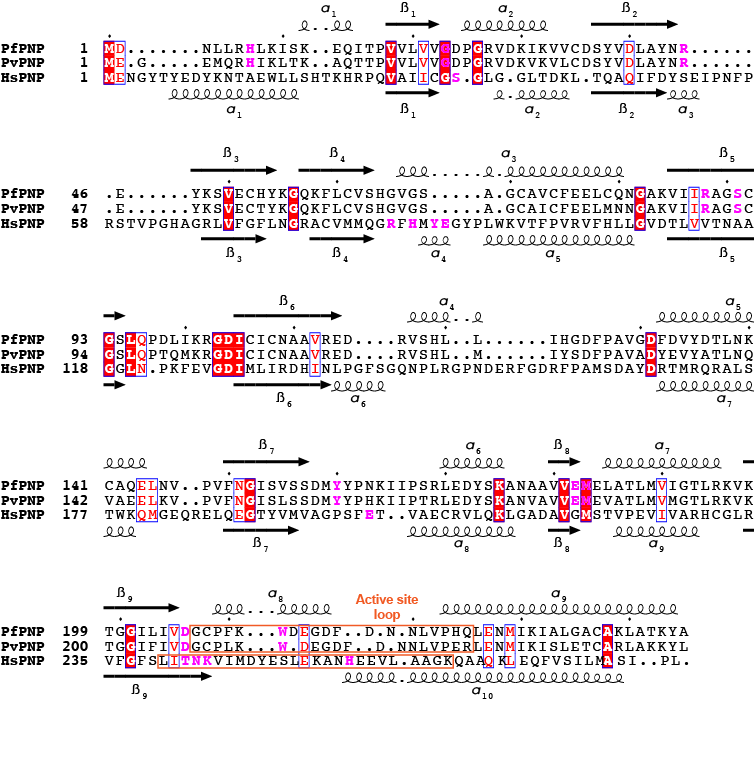


**Supplementary Figure 4. Alignment of amino-acid sequences of PNP from *Plasmodium falciparum* (*Pf*PNP), *Plasmodium vivax* (*Pv*PNP) and from *Homo sapiens* (*Hs*PNP).** The alignment is based on a structure overlay using the TM-align server (https://zhanggroup.org/TM-align/). The overall amino-acid sequence identity between *Pf*PNP and *Pv*PNP (PDB code: 3EMV) is 82 % (with an r.m.s deviation of 1.42 Å for 239 superimposed residues), while it is only 9% between *Pf*PNP and *Hs*PNP (PDB code: 1RCT). Evolutionary-conserved residues are in white, highlighted in red boxes, while similar residues are boxed in blue. Secondary structure elements are indicated for *Pf*PNP and *Pv*PNP (above the sequence) and *Hs*PNP (below the sequences). Active-site residues of PNP are coloured in magenta. The active site loop is boxed in orange (see text).


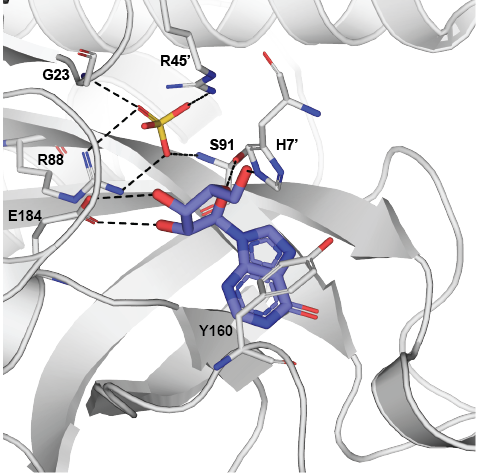


**Supplementary Figure 5. Active site of *Pf*PNP with inosine (slate sticks) and sulfate ion (yellow sticks) bound.** Structures from *Pf*PNP-Inosine (PDB code: 2BSX) and *Pf*PNP-SO_4_ (PDB code: 1SQ6) were superimposed to highlight *Pf*PNP residues contacting the substrate prior to phosphorylation.


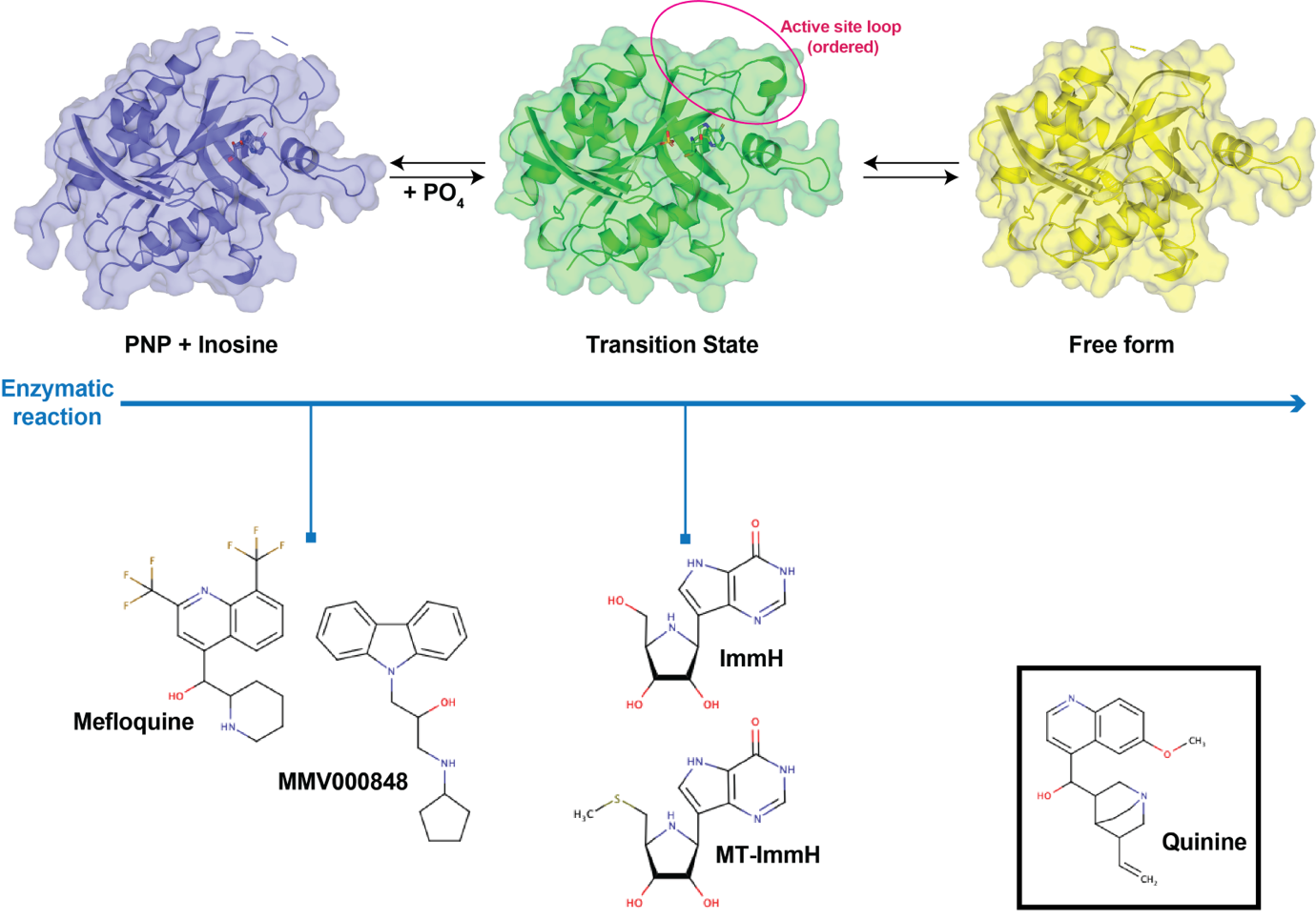


**Supplementary Figure 6. Structures of *Pf*PNP inhibitors mentioned in the text and possible stages of the enzymatic reaction at which inhibition occurs.** Top panel shows surface representations for the pre-reaction state with *Pf*PNP bound to inosine (PDB code: 2BSX) shown in slate. The transition state intermediate (PDB code: 1NW4) bound to ImmH and sulfate is colored in green. The final stage at which products are released is colored in yellow (Free form). Chemical structures of MMV000848, quinoline compounds mefloquine and quinine and immucillin transition state analogue mentioned in the text are depicted in the bottom panel. Mefloquine and MMV000848 appear to stabilise a pre-transition state conformation of *Pf*PNP, while ImmH and MT-ImmH stabilise the transition state intermediate.

|  | MMV000848 |
| --- | --- |
| IC_50_ (µM) | 21.49 |
| Std. Error IC_50_ | 2.357 |
| 95% CI IC_50_ | 17.11 to 26.99 |
| R squared | 0.9878 |
| K_M_ (µM) | 5.712 |
| Std.Error K_M_ | 0.6730 |
| 95% CI K_M_ | 4.410 to 7.214 |
| R squared | 0.9669 |
| K_i_ (µM) | 0.4549 |
| Std.Error K_i_ | 1.447 |
| 95% CI K_i_ | 0.3509 to 0.5790 |
| R squared | 0.9669 |

**Supplementary Table 1.** *Pf*PNP kinetic parameters derived from the inhibition assay with MMV000848.

| **Complex** | **Ligand/Ion** | **Amino acid residue** | **Type of interaction** |
| --- | --- | --- | --- |
| **PfPNP-MMV000848** | **MMV000848** | Tyr160 | Van der Waal’s interactions |
|  |  | Ser91 | Water-mediated hydrogen bond |
|  |  | Val181 | Van der Waal’s interactions |
|  |  | Glu184 | Hydrogen bond |
|  |  | Asp206 | Water-mediated hydrogen bond |
|  | **Phosphate** | Gly23 | Hydrogen bond |
|  |  | Arg27 | Hydrogen bond |
|  |  | Arg88 | Hydrogen bond |
|  |  | Ser91 | Hydrogen bond |
|  |  | Glu184 | Water-mediated hydrogen bond |
|  |  | Arg45’ | Hydrogen bond |
| **PfPNP-Quinine** | **Quinine** | Tyr160 | Van der Waal’s interactions |
|  |  | Glu184 | Water-mediated hydrogen bond |
|  |  | Asp218 | Hydrogen bond |
|  |  | Trp212 | Water-mediated hydrogen bond |
|  | **Phosphate** | Gly23 | Hydrogen bond |
|  |  | Gly65 | Water-mediated hydrogen bond |
|  |  | Arg88 | Hydrogen bond |
|  |  | Ser91 | Hydrogen bond |
|  |  | Glu184 | Water-mediated hydrogen bond |
|  |  | Arg45’ | Hydrogen bond |
| **PfPNP-Mefloquine** | **Mefloquine** | Tyr160 | Van der Waal’s interaction |
|  |  | Glu184 | Water-mediated hydrogen bond |
|  |  | Ser91 | Water-mediated hydrogen bond |
|  |  | Arg88 | Water-mediated hydrogen bond |
|  | **Phosphate** | Ser91 | Hydrogen bond |
|  |  | Arg88 | Hydrogen bond |
|  |  | Glu184 | Water-mediated hydrogen bond |
|  |  | Arg45’ | Hydrogen bond |

**Supplementary Table 2. Summary of interactions observed between *Pf*PNP and MMV000848 (PDB code: 8W7H, this work) or with quinoline inhibitors.** PDB codes: 5ZNC (PfPNP with quinine) and 5ZNI (PfPNP with mefloquine), see reference 32**.** Residues making contact with the inhibitor emanating from the *Pf*PNP dimeric partner are indicated with a prime.

**Video 1. Overall view of the *Pf*PNP hexamer.** Each monomer is represented as ribbons with two-fold symmetrical dimers in light and dark color. Ligands phosphate and MMV000848 are represented as sticks and bound water molecules as blue spheres.

**Video 2. Close-up view of the MMV000848 binding site.** A view of the *Pf*PNP monomer depicted as pink ribbons with a transparent surface, bound to the MMV000848 inhibitor (blue sticks) and the phosphate bound (yellow sticks) in the active site. This is followed by a magnified view of atomic interactions (represented as dashes). Nitrogen atoms are in dark blue, oxygen atoms: red. Red spheres depict water molecules oxygen atoms.
